# Significance testing of rank cross-correlations between autocorrelated time series with short-range dependence

**DOI:** 10.1080/02664763.2022.2137115

**Published:** 2022-10-28

**Authors:** David Lun, Svenja Fischer, Alberto Viglione, Günter Blöschl

**Affiliations:** a Institute of Hydraulic Engineering and Water Resources Management, Vienna University of Technology, Vienna, Austria; b Institute of Hydrology, Water Resources Management and Environmental Engineering, Ruhr-University Bochum, Bochum, Germany; c Department of Environment, Land and Infrastructure Engineering, Politecnico di Torino, Turin, Italy

**Keywords:** Spearman’s Rho, Kendall’s Tau, autocorrelation, β-mixing, significance testing

## Abstract

Statistical dependency measures such as Kendall’s Tau or Spearman’s Rho are frequently used to analyse the coherence between time series in environmental data analyses. Autocorrelation of the data can, however, result in spurious cross correlations if not accounted for. Here, we present the asymptotic distribution of the estimators of Spearman’s Rho and Kendall’s Tau, which can be used for statistical hypothesis testing of cross-correlations between autocorrelated observations. The results are derived using U-statistics under the assumption of absolutely regular (or *β*-mixing) processes. These comprise many short-range dependent processes, such as ARMA-, GARCH- and some copula-based models relevant in the environmental sciences. We show that while the assumption of absolute regularity is required, the specific type of model does not have to be specified for the hypothesis test. Simulations show the improved performance of the modified hypothesis test for some common stochastic models and small to moderate sample sizes under autocorrelation. The methodology is applied to observed climatological time series of flood discharges and temperatures in Europe. While the standard test results in spurious correlations between floods and temperatures, this is not the case for the proposed test, which is more consistent with the literature on flood regime changes in Europe.

## 1. Introduction

Nonparametric measures of association between random variables such as Kendall’s Tau τ and Spearman’s Rho $\rho_{S}$ are frequently used to investigate dependencies in environmental data analyses [45,21,25,46,37]. They have convenient properties: They are invariant under monotone transformations, depending only on the joint behaviour of the random variables as captured by their copula [47]. What is often desired in statistical analyses of environmental data are statistical significance tests to assess if an estimated statistical relationship between observations is due to random chance. The distribution of Kendall’s Tau and Spearman’s Rho for testing the significance of cross-correlations is well understood for independent and identically distributed (iid) random variables [22].

**Figure 1.**
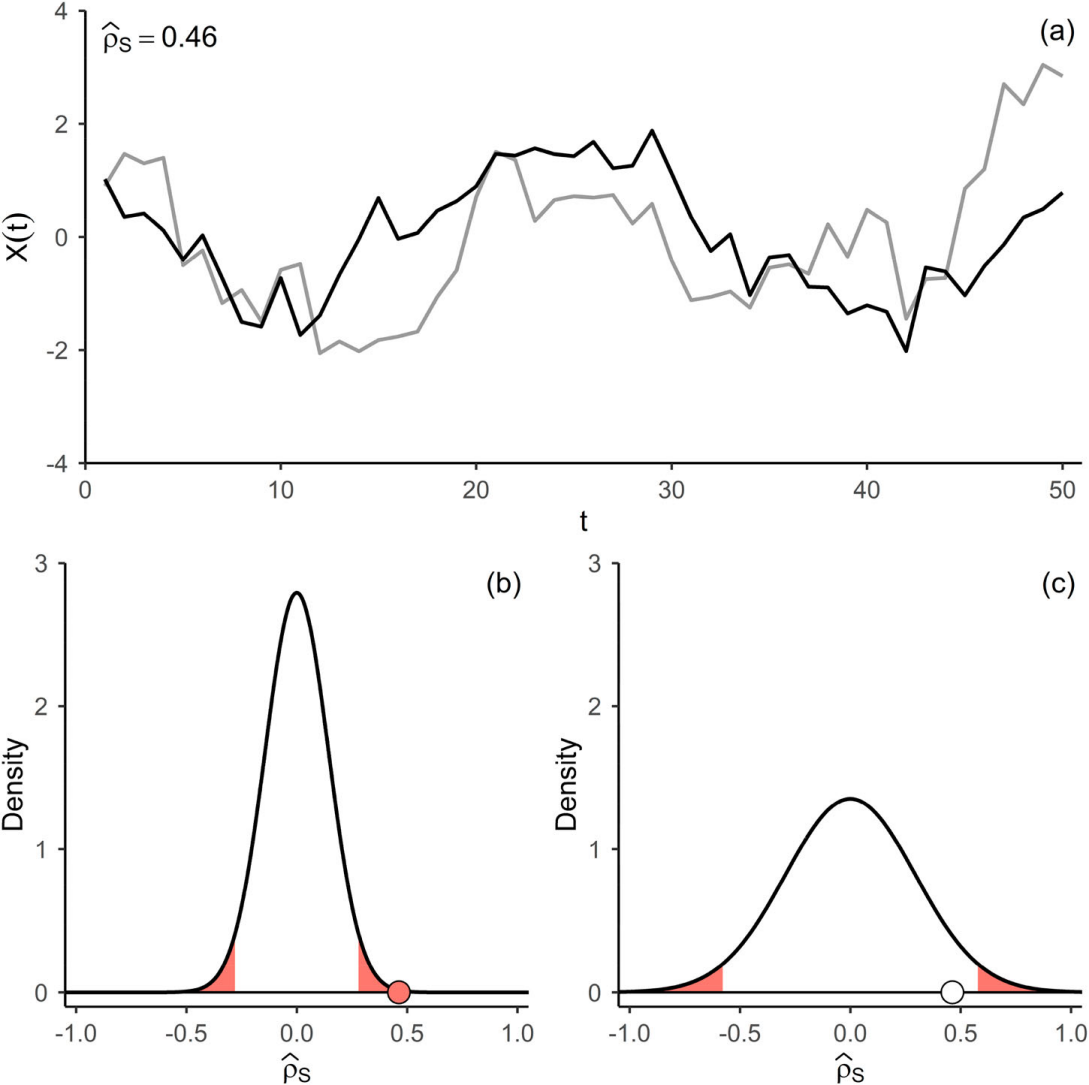
(a) Realization of a bivariate VAR(1)-process $X_{t}$ with Gaussian noise, normal marginal distributions, individual AR(1)-parameters of 0.8, but no dependence between the components. Estimate of Spearman’s Rho for the sample at the top left of the panel. (b) Asymptotic distribution of the estimator of Spearman’s Rho $\rho_{S}$ under $H_{0}$ (no pairwise dependence) for independent observations. (c) Asymptotic distribution of the estimator of Spearman’s Rho under $H_{0}$ for dependent observations (see Corollary 2.1). In panels (b) and (c) the critical region for the corresponding significance test at α=0.05 are highlighted, and the sample-estimate of Spearman’s Rho for the trajectory in panel (a) is depicted as a circle.

However, the assumption of independent observations is often unrealistic for environmental data. Autocorrelation in observations results in higher sampling uncertainty for the statistical estimation of parameters [13,30,32,51]. To assess the statistical dependence between autocorrelated observations via a hypothesis test, the corresponding test statistic needs to be adjusted, i.e. a different limiting distribution is required in the testing procedure. It is understood that for positive autocorrelations the variance of the test statistics is inflated [24,16]. That is because autocorrelation in stochastic processes tends to result in realizations with patterns not occurring at all, or very rarely, for processes that are only comprised of independent noise [31,13]. The peculiarities of the patterns depend on the type of stochastic process and its dependence structure. Realizations of bivariate random variables that are pairwise statistically independent but individually autocorrelated, sometimes result in patterns, which can be mistakenly interpreted as dependence. This is illustrated in Figure 1.

In panel (a) of Figure 1 a realization of a bivariate Vector Autoregressive Process of order 1 (VAR(1)) with Gaussian noise and standard-normal marginals is depicted. The marginals are statistically independent from each other, but are individually autocorrelated. The realization conveys a pattern that could suggest dependence between the components as their trajectories seem to align. The corresponding estimate of Spearman’s Rho for the bivariate sample is roughly 0.46. Figure 1(b) depicts the asymptotic sampling distribution of the estimator for Spearman’s Rho for iid observations, where the areas shaded in red depict the critical region of the corresponding hypothesis test. For the sample in panel (a) the test would result in a rejection of the Null Hypothesis. Panel (c) depicts the asymptotic sampling distribution of the estimator accounting for autocorrelation in the components proposed in this paper (see Corollary 2.1), resulting in a larger variance of the distribution and no rejection of the Null Hypothesis for the sample in panel (a), as the estimate of Spearman’s Rho is outside the critical region. Repeating this simulation exercise 10,000 times for a sample size of 50, with parameters as stated above, results in approximately 31% rejections of the Null Hypothesis of pairwise independence at a significance level of α=0.05, when autocorrelation is not accounted for, corresponding to an inflation of the type-1 error rate of the test. Using a distribution accounting for the presence of autocorrelation in the components for the hypothesis test maintains the prescribed nominal rate of type 1 errors.

In this paper we present the asymptotic distribution of the estimators of Spearman’s Rho and Kendall’s Tau for bivariate random variables, that are pairwise independent, but individually autocorrelated. The result can be used to account for autocorrelation in hypothesis tests of cross-correlations. The asymptotic distribution is derived under the assumption of a strictly stationary, absolutely regular (or β-mixing) discrete-time stochastic process. We also show the consistency of an estimator for the long-run variance of the test statistics and the consistency of the test itself. Section 2 presents the main results as well as common stochastic models in the environmental sciences to which they apply. Proofs can be found in the supplementary material. Section 3 is split into two parts and contains simulation studies investigating the size and power of the suggested hypothesis test for small to moderate sample sizes. In the second part, the modified test is applied to smoothed time series of annual flood peaks and temperatures from the European data set of [4]. The procedure accounting for autocorrelation yields results that are consistent with the literature on flood regime changes on the European scale.

## 2. Methodology

### 2.1. Main results

For a bivariate random variable Spearman’s Rho $\rho_{S}$ and Kendall’s Tau τ are measures of dependence that quantify the strength of monotonic relationships. They only depend on the corresponding copula of the joint distribution and are independent from the marginal distributions of the components of the bivariate random variable [47]. For a given random sample $(X_{i},Y_{i}{)}_{1\leq i\leq n}$, they can be estimated using the ranks $R_{i}$ ( $R_{i}$ is the rank of the *i*-th observation, with rank 1 corresponding to the smallest observation). Their most widely-used estimators are given by equations (1.1) and (1.2).

(1.1) $$\begin{array}{r} {\hat{\rho }}_{S}=\frac{\sum_{j=1}^{n}(R_{j}^{X}-{\bar{R}}^{X})(R_{j}^{Y}-{\bar{R}}^{Y})}{\sqrt{\sum_{j=1}^{n}{\left(R_{j}^{X}-{\bar{R}}^{X}\right)}^{2}\sum_{j=1}^{n}{\left(R_{j}^{Y}-{\bar{R}}^{Y}\right)}^{2}}} \end{array}$$

(1.2) $$\begin{array}{r} \hat{\tau }=\frac{2\sum_{1\leq i<j\leq n}sgn(R_{j}^{X}-R_{i}^{X})sgn(R_{j}^{Y}-R_{i}^{Y})}{n(n-1)} \end{array}$$

Here, sgn refers to the sign-function. These rank correlation measures are often preferred over the classical Pearson correlation in environmental data analyses, due to their robustness, their suitability for heavy-tailed distributions and their ability to capture monotonic dependencies in addition to linear dependencies, which are important properties for the investigation of time series in environmental data analyses [26,45,25]. In addition to cross-correlations, Spearman’s Rho and Kendall’s Tau are also used for detecting autocorrelation in time series [19].

Our results concern strictly stationary, absolutely regular stochastic processes, which comprise a wide variety of stochastic processes used in the environmental sciences.

**Definition 2.1**: Let (Ω,F,P) be a probability space and A and B be two σ-fields. The absolute regularity (or β-mixing) coefficient is defined as

(2.1) $$\beta (A,B)=\sup\frac{1}{2}\overset{I}{\underset{i=1}{\sum }}\overset{J}{\underset{j=1}{\sum }}|P(A_{i}\cap B_{j})-P(A_{i})P(B_{j})|$$

where the supremum is taken over all pairs of finite partitions $\left\{A_{1},\ldots ,A_{I}\right\}$ and $\left\{B_{1},\ldots ,B_{J}\right\}$ of Ω with $A_{i}\in A$ for i=1,…,I and $B_{j}\in B$ for j=1,…,J. Let $(Z{)}_{i\in Z}$ be a p-variate strictly stationary stochastic process on (Ω,A,P). For a≤b let $F_{a}^{b}=\sigma (Z_{a},\ldots ,Z_{b})$ be the σ-field generated by $\left\{Z_{a},\ldots ,Z_{b}\right\}$. The process $(Z{)}_{i\in Z}$ is called absolutely regular (or β-mixing), if

(2.2) $$\beta_{k}=\beta (F_{-\infty }^{0},F_{k}^{\infty })$$

converges to 0 as k→∞.

The β-mixing coefficient is a measure of dependence between σ-fields and lies between 0 and 1, where 0 corresponds to independence. β-mixing of stochastic processes refers to the σ-fields generated by components of the process. β-mixing is a stronger mixing condition than strong (or α-)mixing. Such mixing conditions are often referred to as short-range dependence (SRD) due to the fast decay of the autocorrelation. For a more detailed description of absolute regularity and other mixing conditions see e.g. [6,7].

**Corollary 2.1**: *Let* $(X_{i},Y_{i}{)}_{i\in Z}$ *be a bivariate, strictly stationary, absolutely regular process with absolutely continuous marginal distributions and* β*-mixing coefficients* $\beta_{k}$ *satisfying*

(3.A) $$\overset{\infty }{\underset{k=1}{\sum }}k\cdot \beta_{k}^{\delta /(2+\delta )}<\infty$$

*for some* δ>0*. Under the assumption of independence between* $(X_{i}{)}_{i\in Z}$ *and* $(Y_{i}{)}_{i\in Z}$*, the limiting distributions of the estimators of Spearman’s Rho* ${\hat{\rho }}_{S}$ *and Kendall’s Tau* $\hat{\tau }$ *between* $(X_{i}{)}_{i\in Z}$ *and* $(Y_{i}{)}_{i\in Z}$ *are given by*

(3.1) $$\begin{array}{r} \sqrt{n}{\hat{\rho }}_{S}\overset{D}{\to }N\left(0,1\;+\;2\underset{j>0}{\sum }\rho_{S}^{X}(j)\rho_{S}^{Y}(j)\right) \end{array}$$

(3.2) $$\begin{array}{r} \sqrt{n}\hat{\tau }\overset{D}{\to }N\left(0,\frac{4}{9}\left(1\;+\;2\underset{j>0}{\sum }\rho_{S}^{X}(j)\rho_{S}^{Y}(j)\right)\right) \end{array}$$

*where* $\rho_{S}^{X}(j)$ *refers to the Spearman-correlation between* $X_{t}$ *and* $X_{t-j}$*, and the analogue applies to* $\rho_{S}^{Y}(j)$*.*

Corollary 2.1 also holds for lagged cross-correlations. The asymptotic variances of the estimators are very similar, with the variance for the estimator of Kendall’s Tau being smaller than that for Spearman’s Rho. The asymptotic distribution of ${\hat{\rho }}_{S}$ mirrors that of Pearson’s correlation coefficient for pairwise independent, but autocorrelated observations, see e.g. equation 11.3.5 in [15] or Theorem 11.2.2. in [8]. The variance of the estimators is inflated when both time series are autocorrelated, but not affected when at most one component is autocorrelated, in which case it simplifies to the expression for iid observations (see e.g. Sections 11.2. and 11.3. in [22]). The degree of inflation depends on the magnitude and speed of decay of the autocorrelations of the components, as captured by the sum of their cross-product. If negative autocorrelations are present, the asymptotic variance can be smaller than in the case of independent observations. For a statistical hypothesis test, a consistent estimator of the long-run variance is required, which is provided by Corollary 2.2.

Corollary 2.2: *Let* $(X_{i},Y_{i}{)}_{i\in Z}$ *be a bivariate, strictly stationary, absolutely regular process with absolutely continuous marginal distributions and* β*-mixing coefficients* $\beta_{k}$ *satisfying equation (3.A). Let* κ *be a kernel function satisfying Assumption 1 in* [29] *(see supplementary material) and* $b_{n}$ *be a non-decreasing sequence with* $b_{n}\to \infty$ *and* $b_{n}=o(n^{1/2})$*. Let* κ *and* $b_{n}$ *also satisfy*

(4.A) $$\overset{n}{\underset{j=1}{\sum }}\sqrt{j}\cdot \kappa \left(\frac{j}{b_{n}}\right)=o(n^{1/2})$$

Then

(4.1) $${\hat{\sigma }}^{2}=1\;+\;2\overset{n-2}{\underset{h=1}{\sum }}\kappa \left(\frac{h}{b_{n}}\right){\hat{\rho }}_{S}^{X}(h){\hat{\rho }}_{S}^{Y}(h)\overset{P}{\to }\;1\;+\;2\underset{h>0}{\sum }\rho_{S}^{X}(h)\rho_{S}^{Y}(h)=\sigma^{2}$$

(4.2) $${\hat{\rho }}_{S}^{X}(h)=\frac{\sum_{i=1}^{n-h}(R_{i}^{X}-{\bar{R}}^{X})(R_{i+h}^{X}-{\bar{R}}^{X})}{\sqrt{\sum_{i=1}^{n}{\left(R_{i}^{X}-{\bar{R}}^{X}\right)}^{2}\sum_{i=1}^{n}{\left(R_{i}^{X}-{\bar{R}}^{X}\right)}^{2}}}$$

(4.3) $${\hat{\rho }}_{S}^{Y}(h)=\frac{\sum_{i=1}^{n-h}(R_{i}^{Y}-{\bar{R}}^{Y})(R_{i+h}^{Y}-{\bar{R}}^{Y})}{\sqrt{\sum_{i=1}^{n}{\left(R_{i}^{Y}-{\bar{R}}^{Y}\right)}^{2}\sum_{i=1}^{n}{\left(R_{i}^{Y}-{\bar{R}}^{Y}\right)}^{2}}}$$

With the estimator from equation (4.1), a hypothesis test for testing the significance of rank cross-correlations between time series can be applied, without explicitly specifying the dependence structure of the data-generating process. The estimator of the long-run variance uses a kernel function κ(.) that maps its inputs to the interval [−1,1]. Its purpose is to put more weight on autocorrelations for small lags, as these autocorrelations can be estimated with higher accuracy than autocorrelations for large lags. Together with the bandwidth, which is a function of the sample size, the kernel function has to fulfil some regularity conditions, which can be found in [29] and equation 4.A, in order to achieve consistency of the long-run variance estimator. The matrices of estimated autocorrelations generated by the estimators from equations 4.2 and 4.3 are positive semidefinite [40]. Finally, the consistency of the hypothesis test is guaranteed by Corollary 2.3.

**Corollary 2.3**: *Let* $(X_{i},Y_{i}{)}_{i\in Z}$ *be a bivariate, strictly stationary, absolutely regular process with absolutely continuous marginal distributions and* β*-mixing coefficients* $\beta_{k}$ *satisfying equation (3.A). Let* κ *be a kernel function satisfying Assumption 1 in* [29] *and* $b_{n}$ *be a non-decreasing sequence with* $b_{n}\to \infty$ *and* $b_{n}=o(n^{1/2})$*. Let* κ *and* $b_{n}$ *also satisfy equation (4.A). Under the assumption of pairwise dependence between* $(X_{i}{)}_{i\in Z}$ *and* $(Y_{i}{)}_{i\in Z}$ *with* $\rho_{s},\tau \neq 0$*, the test based on the test statistics*

(5.1) $$\begin{array}{r} T_{\rho_{s}}=\frac{\sqrt{n}{\hat{\rho }}_{S}}{{\hat{\sigma }}^{2}} \end{array}$$

(5.2) $$\begin{array}{r} T_{\tau }=\frac{\sqrt{n}\hat{\tau }}{\frac{4}{9}{\hat{\sigma }}^{2}} \end{array}$$

*with* ${\hat{\sigma }}^{2}$ *from equation (4.1) is consistent.*

Proofs for all Corollaries in this section are provided in the supplementary material.

### 2.2. Examples of *β*-mixing time series models in environmental applications

There is a wide class of time series models in environmental applications that fulfil the conditions of Corollary 2.1. ARMA-models are among the most popular stochastic models for time series analysis in the environmental sciences [50,33,34,45,37]. Weakly stationary ARMA processes are β-mixing if the innovations are absolutely continuous random variables [44]. In this case, the β-mixing coefficients decay geometrically: $\beta_{k}=O(\rho^{k})$ for some 0<ρ<1. This also guarantees that the summability condition in equation (3.A) holds. [9] give necessary and sufficient conditions for the strict stationarity of ARMA processes. Other modelling approaches in environmental data analyses include GARCH processes (e.g. [49,43]). GARCH processes are also absolutely regular under certain conditions (see [10], especially table 1 and Proposition 12). [10] also give sufficient conditions for the strict stationarity of various GARCH processes. Again, under these conditions the decay of the β-mixing coefficients is exponential and equation (3.A) holds.

Other common modelling approaches include Gaussian processes with parametrized autocorrelation functions [48]. Often realizations of these processes are transformed for modelling purposes, for example via quantile-to-quantile transformations, to obtain a desired marginal distribution. Assuming β-mixing of the parent Gaussian process, mixing conditions for these transformations are preserved, as measurable functions (in this case cumulative distribution functions and their inverses) of mixing processes result in mixing processes [5]. The mixing coefficients of the transformed process are smaller or equal to the mixing coefficients of the parent Gaussian process [5]. Sufficient conditions for β-mixing of a discrete-time Gaussian process can be found in [28] (see Theorem 8 and Lemma 6 in Chapter 4.4). These conditions are related to the spectral density of the process and can be verified for a given autocorrelation structure. The asymptotic mixing rate, the speed of decay of $\beta_{k}$, can be obtained from Theorem 4.2 in [53]. For instance: For a stationary, discrete-time Gaussian process $\text{Cov}(X_{0},X_{k})=O(\rho^{k})$ for some 0<ρ<1 yields $\beta_{k}=O(\rho^{k})$. $\text{Cov}(X_{0},X_{h})=O(k^{-\gamma })$ with γ>2 yields $\beta_{k}=O(k^{2-\gamma })$.

M-dependent processes, such as finite-order Moving Average processes, are also common stochastic models in environmental applications. M-dependent processes are β-mixing and equation (3.A) always holds. However, β-mixing processes do not include long-range dependent processes such as Fractional Gaussian noise [38], which is also used for modelling environmental data [31,32].

The assumptions on the kernel function in Corollary 2.2 and 2.3 are satisfied by a large number of kernels, such as the Bartlett-kernel $\kappa (t)=(1-|t|)I_{\left[-1,1\right]}(t)$ and the quartic kernel $\kappa (t)=(1-t^{2}{)}^{2}I_{\left[-1,1\right]}(t)$. The bandwidth $b_{n}$ needs to be chosen so that equation (4.A) is satisfied.

## 3. Application

In this section we assess the performance of the testing procedures for rank cross-correlations based on the results from Section 2.1 for small to moderate sample sizes and compare it with the conventional testing procedure that does not account for autocorrelations for two widely used stochastic models: A VAR(1)-model and finite Moving Averages of independent innovations, corresponding to smoothed time series. Subsequently we apply the testing procedures to smoothed time series of temperatures and discharges and interpret the results.

### 3.1. Simulation studies

We compare the procedure based on the results from Section 2.1, which we refer to as the ‘modified test’, and the procedure assuming the bivariate stochastic process $(X_{i},Y_{i}{)}_{i\in Z}$ is iid, which we refer to as the ‘classical test’. We only present results for Spearman Rho, as the results for Kendall’s Tau are fairly similar. We use the test statistic

(6.1) $$T_{\rho_{s}}=\frac{\sqrt{n}{\hat{\rho }}_{S}}{{\hat{\sigma }}^{2}}$$

where ${\hat{\sigma }}^{2}$ is estimated via equation (4.1) for the modified test and set to $\sqrt{n/(n-1)}$ for the classical test. In both cases, the significance is evaluated by using a normal distribution. We use the quartic kernel

(6.2) $$\kappa (t)=(1-t^{2}{)}^{2}I_{\left[-1,1\right]}(t)$$

and choose the bandwidth as $b_{n}=3n^{1/4}$. There are procedures for adaptively choosing a bandwidth based on data which may improve the performance of the testing procedure, but they are not discussed in the present article.

We consider two different stochastic processes in our simulation study. For both models, an iid process can arise as a special case.

(7.1) $$\text{Model }1:\;\;\left(\begin{array}{c} X_{i}\\ Y_{i} \end{array}\right)=\left(\begin{array}{cc} \varphi_{X} & 0\\ 0 & \varphi_{Y} \end{array}\right)\left(\begin{array}{c} X_{i-1}\\ Y_{i-1} \end{array}\right)+\left(\begin{array}{c} \varepsilon_{i}\\ \delta_{i} \end{array}\right),\;\;\;i\in Z$$

(7.2) $$\left(\begin{array}{c} \varepsilon_{i}\\ \delta_{i} \end{array}\right)∼N(0,\Sigma ),\;\;\Sigma =VAR\left(\begin{array}{c} \varepsilon_{i}\\ \delta_{i} \end{array}\right)=\left(\begin{array}{cc} 1 & \rho \\ \rho & 1 \end{array}\right)$$

(7.3) $$\text{Model }2:\left(\begin{array}{c} X_{i}\\ Y_{i} \end{array}\right)=\frac{1}{2q+1}\overset{q}{\underset{j=-q}{\sum }}\left(\begin{array}{cc} 1 & 0\\ 0 & 1 \end{array}\right)\left(\begin{array}{c} \varepsilon_{i+j}\\ \delta_{i+j} \end{array}\right),\;\;i\in Z,\;\;q\in N$$

(7.4) $$\left(\begin{array}{c} \varepsilon_{i}\\ \delta_{i} \end{array}\right)∼t_{v}(0,\Sigma ),\;\;\Sigma =\left(\begin{array}{cc} 1 & \rho \\ \rho & 1 \end{array}\right),\;\;\nu >0$$

Model 1 is a VAR(1)-model where the error term follows a bivariate normal distribution. The parameters $\varphi_{X}$ and $\varphi_{Y}$ determine the autocorrelation of the components of the process. Model 1 becomes an iid process for $\varphi_{X}=\varphi_{Y}=0$. The Spearman cross-correlation between the components of the process is $\rho_{s}=\frac{6}{\pi }\text{asin}\left(\frac{\rho }{2}\right)$ (see e.g. [41]), where the model parameter ρ also equals the Pearson cross-correlation between the components. Model 2 is a Vector Moving Average Process with independent innovations that follow a bivariate t-distribution. The coefficients are chosen so that they sum up to 1 and are equal, and depend on the order of the model. For q=0, we obtain an iid process. The marginal distributions of Model 2 are heavy-tailed. For ν≤2, the Pearson cross-correlation between the marginals is undefined, whereas the Spearman and Kendall cross-correlations are well-defined and finite for all ν>0. Model 2 is a suitable model for smoothed time series, which are frequently encountered in environmental data analyses if the long-term behaviour is of interest. For both models, the marginal distributions of the components can be transformed to any other distribution with absolutely continuous distribution function (via quantile-to-quantile transformations) without affecting the mixing properties of the process, the Spearman correlation between the components and the performance of the testing procedure.

**Figure 2.**
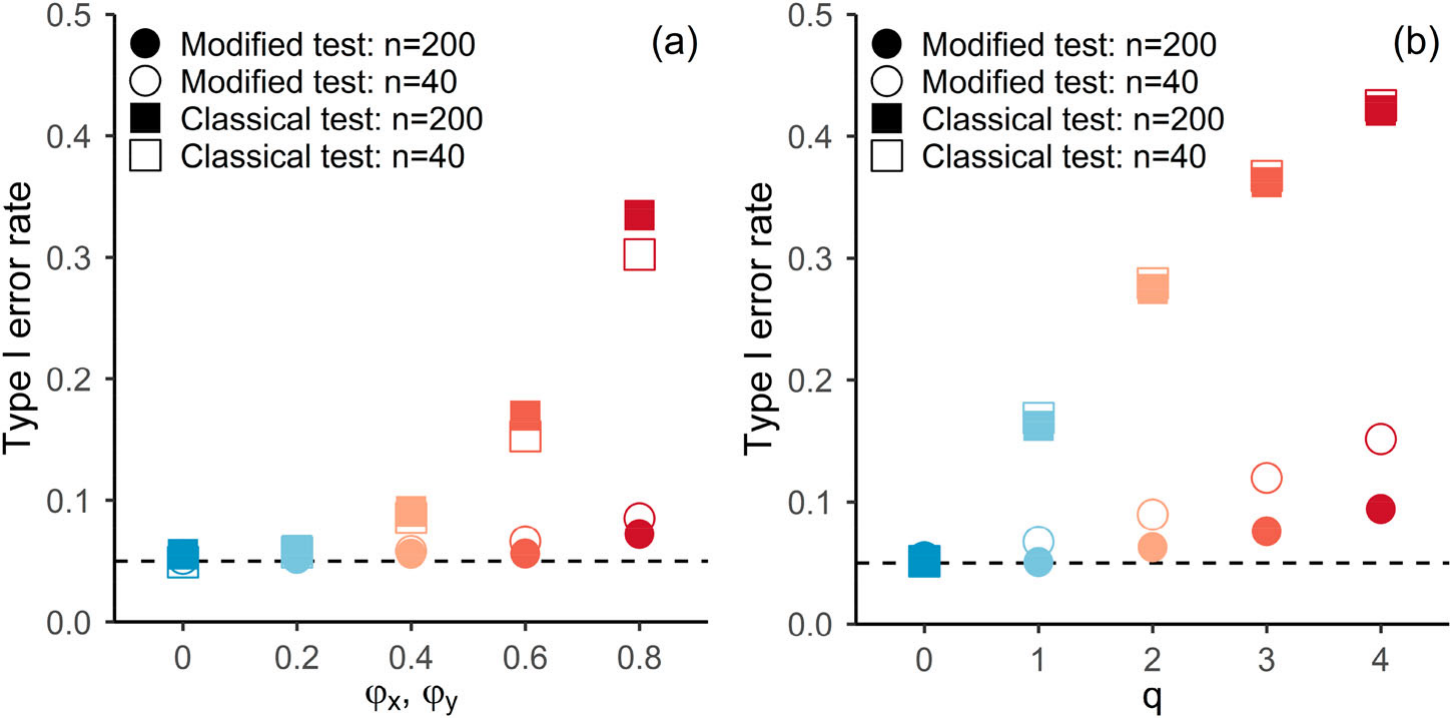
Observed type 1 error rate for two-sided significance test of Spearman’s Rho for Model 1 (panel a) and Model 2 (panel b) at *α* = 0.05 based on simulations (10,000 runs). Horizontal axes represent the parameters of the models governing the autocorrelations of the components ( $\varphi_{X}=\varphi_{Y}$ and ν=4 for all results shown here), shapes indicate which asymptotic distribution was used for the significance test, i.e. Squares: classical test; Circles: modified test. Open symbols: *n* = 40, Full symbols: *n* = 200.

Figure 2 shows the observed frequency of type-1 errors for testing the significance of Spearman cross-correlation between X and Y, as a function of different degrees of autocorrelation, which is determined by the parameters of Model 1 and Model 2 (also indicated by colour), employing a two-sided test at a significance level of *α* = 0.05. The results are based on 10,000 simulations for each parameter configuration shown in the figure. For Model 2, two univariate independent t-distributions were used for the error term instead of a bivariate t-distribution with ρ=0. We show results for two different sample sizes (40 and 200) indicated by open and full symbols. The type 1 error rate of a statistical test usually isn’t overly sensitive to the number of observations, as we control for sample size in the test statistic. However, the adequacy of the asymptotic result (Corollary 2.1), as well as the accuracy of the long-run variance estimate (Corollary 2.2) does depend on the sample size. Sample sizes as small as 40 for individual time series are frequently encountered in the environmental sciences, especially when annual values or extremes are of interest (see e.g. [12,35,23]).

Figure 2 shows that the observed number of rejections under the Null Hypothesis is strongly affected by the presence of autocorrelations. For small sample sizes ( n=40), the modified test gives a slightly larger type 1 error rate than the selected significance level. This is especially noticeable in the presence of strong autocorrelations. However, this effect vanishes with increasing sample size. On the other hand, not accounting for autocorrelations in the components substantially affects the observed type 1 error rate, starting at moderate levels of autocorrelation (e.g. $\varphi_{X}=\varphi_{Y}=0.4$, q=1, orange shapes in Figure 2). In the case of weakly autocorrelated component processes, the resulting type 1 error rate is only slightly elevated, but the modified test is also able to maintain the type 1 error rate in this case (blue shapes in Figure 1).

**Figure 3.**
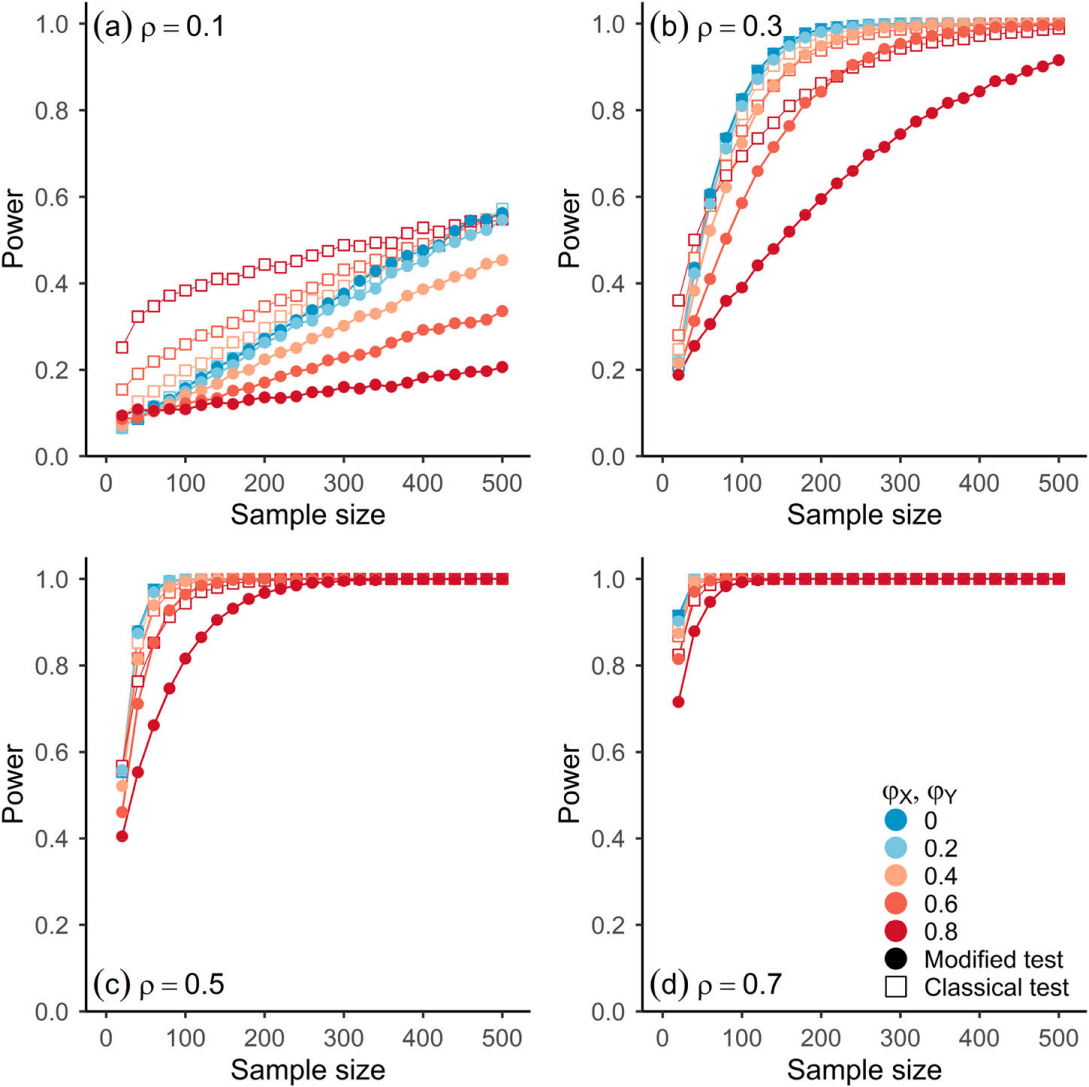
Observed power for two-sided significance tests of Spearman’s Rho for Model 1 (equations 7.1 & 7.2) and different sample sizes at *α* = 0.05 based on simulations (10,000 runs). Panels refer to results for different values of the parameter ρ of Model 1. Horizontal axes represent sample size. $\varphi_{X}=\varphi_{Y}$ for all results shown here. Shapes indicate which asymptotic distribution was used for the significance test, i.e. (Open squares) classical test, (Full circles) modified test.

**Figure 4.**
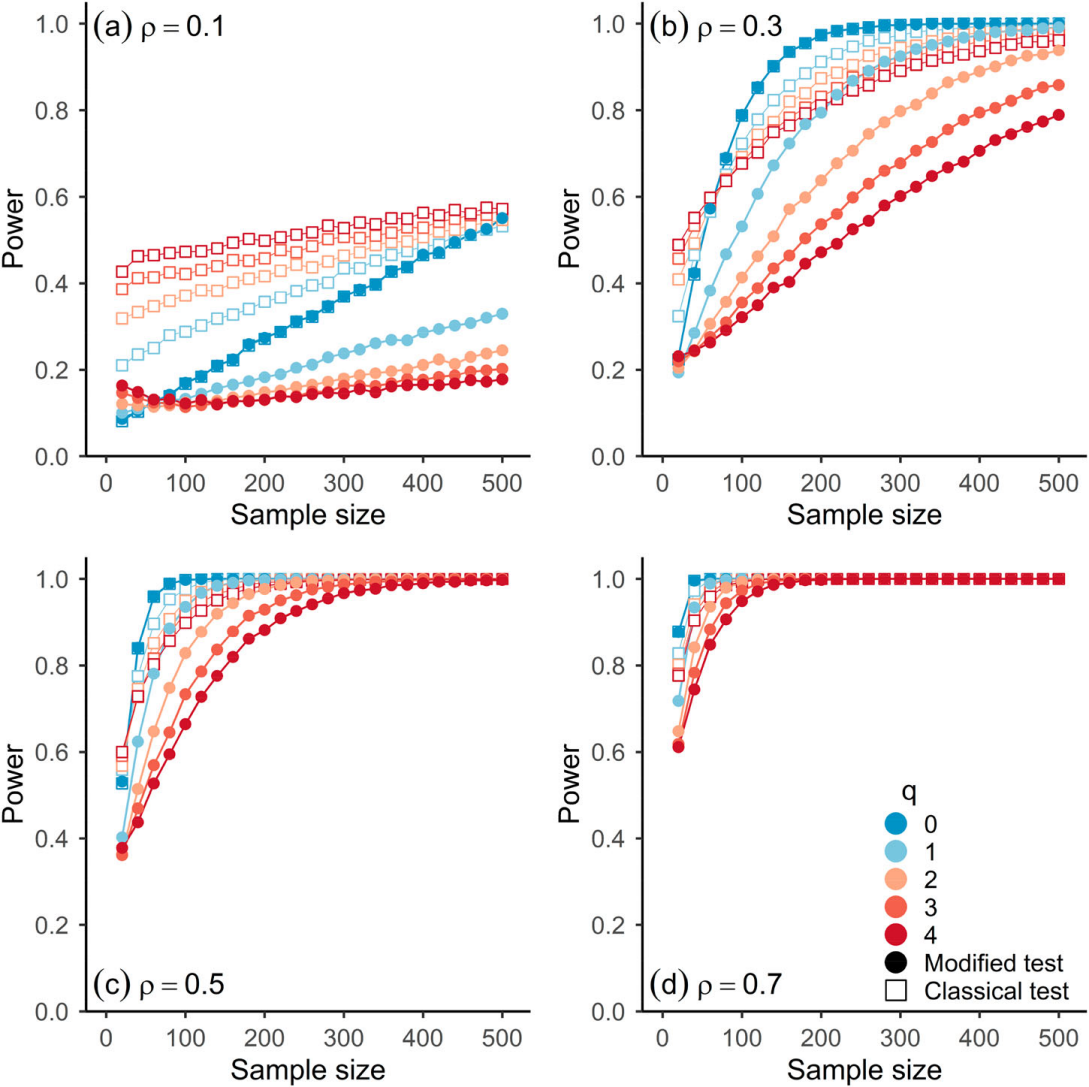
Observed power for two-sided significance tests of Spearman’s Rho for Model 2 (equations 7.3 & 7.4) and different sample sizes at *α* = 0.05 based on simulations (10,000 runs). Panels refer to results for different values of the parameter ρ of Model 2 ( ν=4). Horizontal axes represent sample size. Shapes indicate which asymptotic distribution was used for the significance test, i.e. (Open squares) classical test, (Full circles) modified test.

Figures 3 and 4 show the power of the testing procedures as a function of sample size for different degrees of dependence between the components of the processes of Model 1 and 2, which is parameterized by ρ (see equations 7.1–7.4). Both figures have four panels, corresponding to different degrees of rank cross-correlations between the components, and depict the observed rejection frequencies for different scenarios of autocorrelation of the component processes (indicated by colour) at a significance level of *α* = 0.05. The results are based on 10,000 simulations for each parameter configuration shown in the figures.

As expected, the power is larger for the classical test. In the presence of autocorrelations, more volatility is expected in statistical estimation procedures. When they are accounted for in statistical hypothesis tests, as in the modified test, the power is affected. In the case of the smallest positive autocorrelations considered here, the loss in power for the modified test is rather small (light blue dots in Figures 3 and 4) and for iid observations the difference in power is close to zero (dark blue dots in Figures 3 and 4). For larger autocorrelations, the loss in power is noticeable, especially for small to moderate cross-correlations (orange and red shapes in panels (a) and (b) in Figures 3 and 4): the vertical distance between the open and full red symbols in the top panels of Figures 3 and 4 is comparatively high and also persists for sample sizes up to 500. However, for strong cross-correlations (e.g. ρ≥0.7), the loss in power goes to zero rather quickly with increasing sample size, as the power rapidly tends towards 1 for both procedures.

As shown in the simulation results in Figures 2–4, the modified test gives correct inferences while controlling the rate of type 1 errors to a satisfactory level, even when an estimate of the long-run variance is used that does not assume any specific structure on the underlying statistical model, besides the necessary assumptions on the data-generating process for the results in Corollary 2.1 to hold. The higher power of the classical test comes at the price of an elevated rate of type 1 errors, which can be substantial when strong autocorrelations are present in both component processes (Figure 2). In the case of weak autocorrelations in the components, the modified test gives a negligible loss in power when compared to the classical test (Figures 3 and 4). For iid observations, the loss in power is practically zero. A more conservative testing procedure with a lower rate of type 1 errors (see Figure 2) can be achieved by increasing the bandwidth $b_{n}$, which, however, also reduces the power (not shown).

### 3.2. Application to hydrological data

Spurious dependencies due to autocorrelated observations are relevant in quantitative analyses of environmental data. Sometimes correlations between smoothed time series are investigated in analyses of climatological data [54,17,39,42,27,20]. In the case of yearly observations, the rationale of using smoothed time series is the interest in the joint long-term behaviour of the series rather than their year-to-year variability [54,42]. However, even if the individual observations are independent, the smoothed time series will be autocorrelated, which in turn can lead to spurious cross-correlations. This can be counteracted by using the modified test suggested in this paper.

We present an example from hydrology on the European scale. [4] analyse over 2000 series of annual maximum peak discharges from 33 countries with observations from 1960 to 2010. Annual maximum peak discharges are an indicator of the flood regime at a river cross-section and used for the estimation of design floods in flood risk management and in the evaluation of the impact of climate change on the water cycle (see e.g. [2,52,18]). A trend analysis shows distinct patterns of flood regime changes on the European scale and hydrological drivers of these changes are discussed in [4]. One of the main patterns is a downward trend of flood peaks in Eastern Europe. The authors argue that temperatures have increased all over Europe, but the effects of this increase on flood peaks are especially drastic and relevant in Eastern Europe, where floods are mainly generated by snowmelt (see their Extended Data Figure 6). Rising temperatures have led to less snow cover and, therefore, smaller flood peaks occurring earlier in the year than some decades ago [3]. The decrease in flood peaks and the increase in temperature in Eastern Europe occurs simultaneously on a decadal scale. In order to investigate this relationship more closely, we examine Spearman correlations between series of flood peaks and average annual temperatures for the catchments in the data set of [4]. The flood data can be downloaded from their supplementary materials. Temperature data are annual averages of daily catchment averages of gridded E-OBS data, see [14].

**Figure 5.**
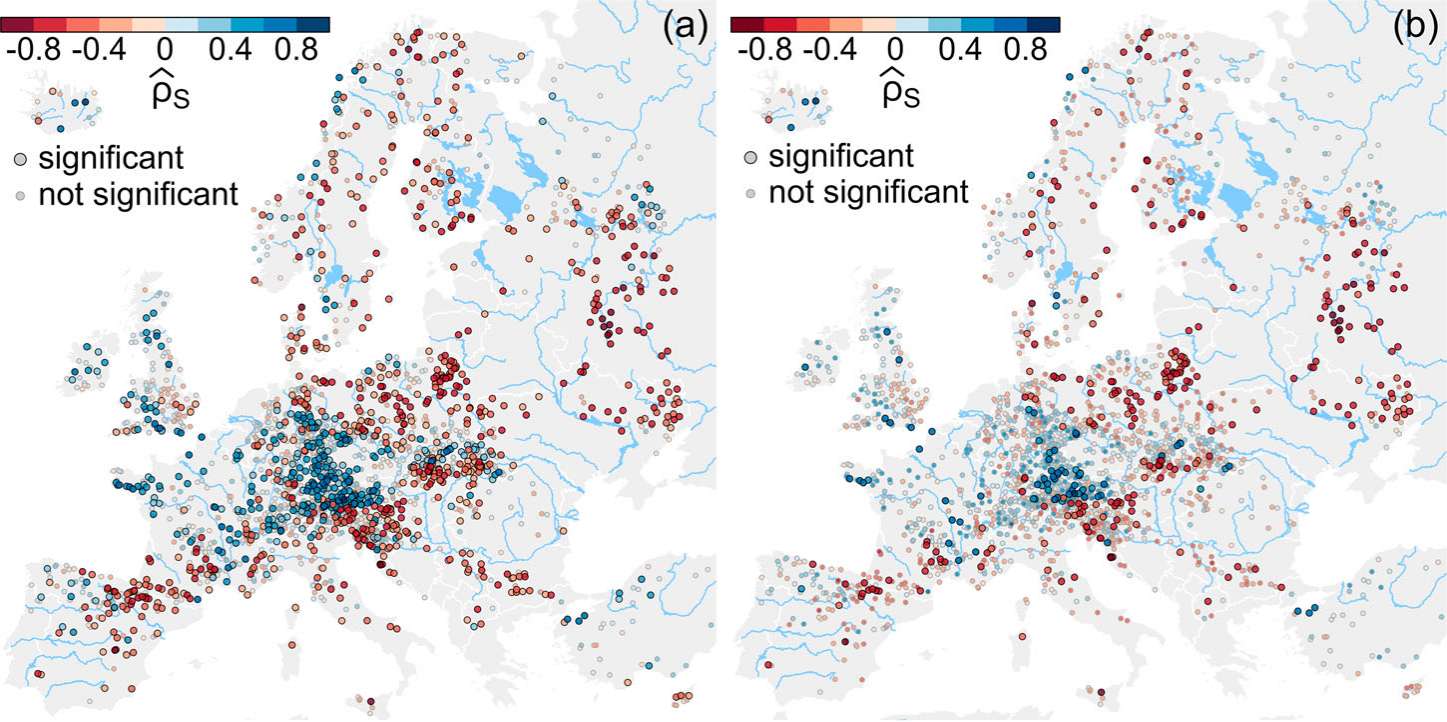
Estimated Spearman-correlations between annual series of flood peak discharges and average annual catchment temperatures. All series are smoothed via a two-sided moving average filter of length 5 with equal weights, centred at the observations (similar to Model 2 with q=2). The circles, representing catchments, indicate the magnitude of the estimated Spearman correlations. The size and the transparency of the circles indicate statistical significance at *α* = 0.05. Panel (a) depicts results of the classical test, panel (b) those of the modified test. Flood data from [4], temperature data from E-OBS, see [14].

We analyse smoothed series of flood peaks and temperatures, as we are interested in their long-term coevolution and whether they are dependent at a multi-annual scale rather than for individual years. We apply a simple two-sided moving average filter of five years with equal weights to the data, centred around the observations. This is similar to Model 2 from section 3.1 with q=2. Annual maxima of flood peaks are modelled with iid random variables in classical flood frequency analysis (see e.g. Chapters 17&18 in [36]). When applying a significance test for cross-correlation, one would expect a significant relationship between flood peaks and temperatures in Eastern Europe, but not so in other regions of Europe, where snow processes (and thus temperature) are much less relevant for flood generation and hence flood changes [4]. Figure 5 shows the estimated Spearman cross-correlation between smoothed series of flood peaks and average annual temperatures.

Figure 5(a,b) show the estimated Spearman correlation between smoothed series of annual flood peaks and annual average temperatures for the classical and the modified significance test, respectively, at a significance level of *α* = 0.05. In panel (a) the tests suggest a statistically significant relationship at roughly half of all locations (1126 out of 2360 stations). When we account for autocorrelation in the individual time series, this number drops drastically, as can be seen in panel (b) (353 out of 2360 stations). Importantly, in the latter case the statistically significant relationships are found almost exclusively for catchments in Eastern and Northern Europe, where snow-processes govern flood behaviour and thus temperature-driven changes are physically very plausible [4]. In the Alps and to a smaller extent in the Ore Mountains some significant positive correlations remain which are also very plausible. Increasing temperature has led to decreasing snowfall limits in these mountainous regions, resulting in more liquid precipitation and increasing flood peaks [1,11]. Overall, when accounting for autocorrelation in significance testing between smoothed series of flood peaks and temperatures, those regions remain significant for which very good physical reasons of such a relationship exist.

## 4. Conclusion

The statistical modelling of autocorrelated observations is associated with increased uncertainty of statistical estimation procedures for parameters compared to the modelling of iid observations and can result in spurious cross-correlations. The use of the asymptotic distributions of estimators of cross-correlations, which account for autocorrelation in the components, can improve the accuracy of statistical inference when dealing with observations with persistence. We presented the asymptotic distribution of the estimators of Spearman’s Rho and Kendall’s Tau under the hypothesis of pairwise independence of the components and β-mixing of the stochastic process, which can be used for statistical hypothesis testing. The modified testing procedure is consistent and simulations show that the procedure also produces satisfactory results for small to moderate sample sizes. Accounting for autocorrelation results in lower statistical power, which is expected. However, the loss in power is negligible when only weak autocorrelations are present in the components and essentially zero for iid observations (Figures 3 and 4). On the other hand, not accounting for autocorrelation in the components does result in elevated rates of type 1 errors, which can be substantial (Figure 2). The suggested testing procedure was applied to 2360 European series of annual maximum flood peak discharges and catchment average annual temperatures. We used smoothed versions of the series, which introduces autocorrelation to the observations, resulting in many spurious correlations between flood peaks and temperature with the standard test. In contrast, with the test proposed here, the plausible locations remain, which are consistent with the literature on flood changes on the European scale. The proposed procedure can be used for analysing any pairs of time series with short-range dependence in the environmental sciences. Possible extensions of the results presented here include confidence intervals for the estimators of Spearman’s Rho and Kendall’s Tau for a β-mixing process, which would require a consistent estimator for the long-run variance under pairwise dependence of the components of the process, or the asymptotic distribution of the estimators for long-range dependent processes.

## Supplementary Material

Supplemental Material

## Data Availability

The flood data used in this paper are available at (https://github.com/tuwhydro/europe_floods). The authors acknowledge the E-OBS dataset from the EU-FP6 project UERRA (http://www.uerra.eu) and the Copernicus Climate Change Service, and the data providers in the ECA&D project (https://www.ecad.eu). An R-Package (https://www.r-project.org/) for the modified testing procedure presented here can be found at (https://cran.r-project.org/web/packages/corTESTsrd/).
