# Supplementary material for: Significance testing of rank cross-correlations between autocorrelated time series with short-range dependence
Source: J Appl Stat. 2022 Oct 28;50(14):2934–50. doi: 10.1080/02664763.2022.2137115 (PMC10557552; doi:10.1080/02664763.2022.2137115)
Supplement: Supplemental Material [file CJAS_A_2137115_SM0992.pdf]

Supplementary material for "Significance testing of rank cross-correlations between autocorrelated time series with short-range dependence"

David Lun[a], Svenja Fischer[b], Alberto Viglione[c] and Günter Blöschl[a]

[a]Institute of Hydraulic Engineering and Water Resources Management, Vienna University of Technology, Vienna, Austria; [b]Institute of Hydrology, Water Resources Management and Environmental Engineering, Ruhr-University Bochum, Bochum, Germany; [c]Department of Environment, Land and Infrastructure Engineering, Politecnico di Torino, Turin, Italy

1 Some preliminary definitions

Spearman's Rho ρ_S and Kendall's Tau τ between two random variables X and Y are defined as

$$\begin{aligned}\rho_S &= 12\iint uvdC(u,v) - 3 = 12\iint F_X(x)F_Y(x)dF_{X,Y}(x,y) - 3 = 12\mathbb{E}\left(F_X(X)F_Y(Y)\right) - 3 \\ &= 12\iint C(u,v)dudv - 3 = 12\iint F_{X,Y}(x,y)dF_X(x)dF_Y(y) - 3 = 12\mathbb{E}_X\mathbb{E}_Y\left(F_{X,Y}(x,y)\right) - 3\end{aligned} \tag{A1.1}$$

$$\tau = 4\iint C(u,v)dC(u,v) - 1 = 4\iint F_{X,Y}(x,y)dF_{X,Y}(x,y) - 1 = 4\mathbb{E}\left(F_{X,Y}(x,y)\right) - 1 \tag{A1.2}$$

Where F_X is the cumulative distribution function (cdf) of X, F_Y is the cdf of Y, $F_{X,Y}$ is the joint cdf of (X,Y) and C is the associated copula of the joint distribution (see e.g. Nelsen, 1992). In the following, we will make use of the concept of U-statistics to provide the limit theorems for the estimators of both correlation measures under short-range dependence. U-statistics average a kernel function over permutations of a subset of a sample, thereby providing an unbiased estimate of a parameter of the underlying distribution. For a symmetric kernel h of degree m, which is just a measurable function symmetrical in its arguments, the corresponding U-statistic can be written as

$$U_n(h) = \frac{1}{\binom{n}{m}} \sum_{1\leq i_1<i_2<\ldots<i_m\leq n} h\left(X_{i_1},\ldots,X_{i_m}\right) \tag{A2}$$

For general theory on U-statistics see e.g. Chapter 12 in van der Vaart (1998) and Dehling (2006).

2 Corollaries, Lemmas and proofs

Corollary 1. *Let $(X_i, Y_i)_{i\in\mathbb{Z}}$ be a bivariate, strictly stationary, absolutely regular process with absolutely continuous marginal distributions and β-mixing coefficients β_k satisfying*

$$\sum_{k=1}^{\infty} k \cdot \beta_k^{\delta/(2+\delta)} < \infty \tag{3.A}$$

for some $\delta > 0$. Under the assumption of independence between $(X_i)_{i\in\mathbb{Z}}$ and $(Y_i)_{i\in\mathbb{Z}}$, the limiting distributions of the estimators of Spearman's Rho $\widehat{\rho}_S$ and Kendall's Tau $\widehat{\tau}$ between $(X_i)_{i\in\mathbb{Z}}$ and $(Y_i)_{i\in\mathbb{Z}}$ are given by

$$\sqrt{n}\widehat{\rho}_S \xrightarrow{D} \mathcal{N}\left(0, 1 + 2\sum_{j>0} \rho_S^X(j)\rho_S^Y(j)\right) \tag{3.1}$$

$$\sqrt{n}\widehat{\tau} \xrightarrow{D} \mathcal{N}\left(0, \frac{4}{9}\left(1 + 2\sum_{j>0} \rho_S^X(j)\rho_S^Y(j)\right)\right) \tag{3.2}$$

where $\rho_S^X(j)$ refers to the Spearman-correlation between X_t and X_{t-j}, and the analogue applies to $\rho_S^Y(j)$.

Proof of Corollary 1. The estimators of Spearman's Rho ρ_S and Kendall's Tau τ can be written as U-statistics. The corresponding symmetric kernels have degree 3 and 2, respectively, and are given by

$$\begin{aligned} h^{\rho_S}\left((X_1,Y_1),(X_2,Y_2),(X_3,Y_3)\right) = 12\Big(&\mathbf{1}_{(0,\infty)}(X_2 - X_1)\mathbf{1}_{(0,\infty)}(Y_3 - Y_1) \\ &+\mathbf{1}_{(0,\infty)}(X_3 - X_1)\mathbf{1}_{(0,\infty)}(Y_2 - Y_1) \\ &+\mathbf{1}_{(0,\infty)}(X_1 - X_2)\mathbf{1}_{(0,\infty)}(Y_3 - Y_2) \\ &+\mathbf{1}_{(0,\infty)}(X_3 - X_2)\mathbf{1}_{(0,\infty)}(Y_1 - Y_2) \\ &+\mathbf{1}_{(0,\infty)}(X_1 - X_3)\mathbf{1}_{(0,\infty)}(Y_2 - Y_3) \\ &+\mathbf{1}_{(0,\infty)}(X_2 - X_3)\mathbf{1}_{(0,\infty)}(Y_1 - Y_3)\Big) - 3 \end{aligned} \tag{A3.1}$$

$$\begin{aligned} h^{\tau}\left((X_1,Y_1),(X_2,Y_2)\right) = 2\Big(&\mathbf{1}_{(0,\infty)}(X_2 - X_1)\mathbf{1}_{(0,\infty)}(Y_2 - Y_1) \\ &+\mathbf{1}_{(0,\infty)}(X_1 - X_2)\mathbf{1}_{(0,\infty)}(Y_1 - Y_2)\Big) - 1 \end{aligned} \tag{A3.2}$$

To obtain the limiting distribution, we make use of the Hoeffding decomposition, more precisely we need the following Hoeffding kernels h_1

$$\begin{aligned} h_1^{\rho_S}\left((x_1,y_1)\right) =& \mathbb{E}h^{\rho_S}\left((x_1,y_1),(X_2,Y_2),(X_3,Y_3)\right) - \rho_S \\ =& 1 - 2F_X(x_1) - 2F_Y(y_1) + 4F_X(x_1)F_Y(y_1) \end{aligned} \tag{A4.1}$$

$$
\begin{aligned}
h_1^{\tau}\left((x_1,y_1)\right) =& \mathbb{E}h^{\tau}\left((x_1,y_1),(X_2,Y_2)\right) - \tau \\
=& 1 - 2F_X(x_1) - 2F_Y(y_1) + 4F_X(x_1)F_Y(y_1)
\end{aligned} \tag{A4.2}
$$

Here the expectation is taken for independent copies of $(X_i, Y_i)_{i\in\mathbb{Z}}$ and assuming independence between $(X_i)_{i\in\mathbb{Z}}$ and $(Y_i)_{i\in\mathbb{Z}}$. The limiting distribution of the estimators can be obtained with Theorem 2 in Dehling (2006). The assumptions of the theorem are met because we have a bounded kernel for both estimators, and we obtain

$$
\sqrt{n}\left(U_n(h) - \theta\right) \xrightarrow{D} \mathcal{N}(0, m^2\sigma^2) \tag{A5.1}
$$

with

$$
\sigma^2 = \mathbb{E}\left(h_1(X_1,Y_1)\right)^2 + 2\sum_{j>1}\mathrm{Cov}\left(h_1(X_1,Y_1), h_1(X_j,Y_j)\right) \tag{A5.2}
$$

Where $U_n(h)$ is the U-statistic estimator of either Spearman's Rho or Kendall's Tau and m is the respective degree of the kernel. For the Hoeffding kernels in equations A4.1 and A4.2, under the assumption of pairwise independence, we obtain

$$
\mathrm{Cov}\left(h_1(X_1,Y_1), h_1(X_j,Y_j)\right) = \mathbb{E}h_1(X_1,Y_1)h_1(X_j,Y_j) = \frac{1}{9}\rho_S^X(j-1)\rho_S^Y(j-1) \tag{A6}
$$

where $\rho_S^X(j-1)$ is the Spearman-autocorrelation of X for lag $j-1$, with $\rho_S^Y(j-1)$ being the analogous for Y. The usual estimator for Kendall's Tau given in equation 1.2 is algebraically equivalent to the U-statistic estimator for Kendall's Tau $U_n(h^{\tau})$; the asymptotic result holds. The algebraic relationship between the estimator for Spearman's Rho in equation 1.1 and the U-statistic for Spearman's Rho is given by

$$
\widehat{\rho}_S = \frac{n-2}{n+1}U_n(h^{\rho_S}) + \frac{3}{n+1}\widehat{\tau} \tag{A7}
$$

It follows that $\sqrt{n}\left(U_n(h^{\rho_S}) - \widehat{\rho}_S\right) = o_P(1)$, therefore the estimators have the same limiting distribution (Theorem 2.7 (iv) in van der Vaart, 1998). □

Corollary 2. *Let $(X_i, Y_i)_{i\in\mathbb{Z}}$ be a bivariate, strictly stationary, absolutely regular process with absolutely continuous marginal distributions and β-mixing coefficients β_k satisfying equation 3.A. Let κ be a kernel function satisfying Assumption 1 (Assumption 1 in de Jong und Davidson (2000)) and b_n be a non-decreasing sequence with $b_n \to \infty$ and $b_n = o(n^{1/2})$. Let κ and b_n also satisfy*

$$\sum_{j=1}^{n} \sqrt{j} \cdot \kappa\left(\frac{j}{b_n}\right) = o(n^{1/2}) \tag{4.A}$$

Then

$$\widehat{\sigma}^2 = 1 + 2\sum_{h=1}^{n-2} \kappa\left(\frac{h}{b_n}\right) \widehat{\rho}_S^X(h)\widehat{\rho}_S^Y(h) \xrightarrow{P} 1 + 2\sum_{h>0} \rho_S^X(h)\rho_S^Y(h) = \sigma^2 \tag{4.1}$$

$$\widehat{\rho}_S^X(h) = \frac{\sum_{i=1}^{n-h}\left(R_i^X - \overline{R}^X\right)\left(R_{i+h}^X - \overline{R}^X\right)}{\sqrt{\sum_{i=1}^{n}\left(R_i^X - \overline{R}^X\right)^2 \sum_{i=1}^{n}\left(R_i^X - \overline{R}^X\right)^2}} \tag{4.2}$$

$$\widehat{\rho}_S^Y(h) = \frac{\sum_{i=1}^{n-h}\left(R_i^Y - \overline{R}^Y\right)\left(R_{i+h}^Y - \overline{R}^Y\right)}{\sqrt{\sum_{i=1}^{n}\left(R_i^Y - \overline{R}^Y\right)^2 \sum_{i=1}^{n}\left(R_i^Y - \overline{R}^Y\right)^2}} \tag{4.3}$$

Assumption 1 (Assumption 1 in de Jong und Davidson (2000))**.** Let $\kappa : \mathbb{R} \to [-1, 1]$ be continuous at 0 and all but a finite number of points and fulfil

$$\kappa(0) = 1$$
$$\kappa(x) = \kappa(-x) \quad \forall x \in \mathbb{R}$$
$$\int_{-\infty}^{\infty} |\kappa(x)|\, dx < \infty$$
$$\int_{-\infty}^{\infty} \left|\frac{1}{2\pi}\int_{-\infty}^{\infty} \kappa(x)e^{i\xi x}dx\right| d\xi < \infty$$

Proof of Corollary 2. Via the dominated convergence theorem it follows that

$$1 + 2\sum_{h=1}^{n-2} \kappa\left(\frac{h}{b_n}\right) \rho_S^X(h)\rho_S^Y(h) \to 1 + 2\sum_{h=1}^{n-2} \rho_S^X(h)\rho_S^Y(h) \tag{A8}$$

The absolute summability of the Spearman-autocorrelations is guaranteed by the assumption on the mixing coefficients. In order to show the consistency of the long-run variance estimator we show that

$$\sum_{h=1}^{n-2} \kappa\left(\frac{h}{b_n}\right) \left(\widehat{\rho}_S^X(h)\widehat{\rho}_S^Y(h) - \rho_S^X(h)\rho_S^Y(h)\right) \xrightarrow{P} 0 \tag{A9}$$

It follows that

$$
\begin{aligned}
&\mathbb{E}\left|\sum_{h=1}^{n-2}\kappa\left(\frac{h}{b_n}\right)\left(\widehat{\rho}_S^X(h)\widehat{\rho}_S^Y(h)-\rho_S^X(h)\rho_S^Y(h)\right)\right| \\
&=\mathbb{E}\left|\sum_{h=1}^{n-2}\kappa\left(\frac{h}{b_n}\right)\left(\widehat{\rho}_S^Y(h)\left(\widehat{\rho}_S^X(h)-\rho_S^X(h)\right)+\rho_S^X(h)\left(\widehat{\rho}_S^Y(h)-\rho_S^Y(h)\right)\right)\right| \\
&\leq \mathbb{E}\sum_{h=1}^{n-2}\kappa\left(\frac{h}{b_n}\right)\left(\left|\widehat{\rho}_S^Y(h)\right|\left|\widehat{\rho}_S^X(h)-\rho_S^X(h)\right|+\left|\rho_S^X(h)\right|\left|\widehat{\rho}_S^Y(h)-\rho_S^Y(h)\right|\right) \\
&\leq \sum_{h=1}^{n-2}\kappa\left(\frac{h}{b_n}\right)\left(\mathbb{E}\left|\widehat{\rho}_S^X(h)-\rho_S^X(h)\right|+\mathbb{E}\left|\widehat{\rho}_S^Y(h)-\rho_S^Y(h)\right|\right) \\
&\leq \sum_{h=1}^{n-2}\kappa\left(\frac{h}{b_n}\right)\left(\mathbb{E}\left|\widetilde{\rho}_S^X(h)-\rho_S^X(h)\right|+\mathbb{E}\left|\widetilde{\rho}_S^Y(h)-\rho_S^Y(h)\right|\right)+C_1\frac{1}{n}\sum_{h=1}^{\lfloor n/2\rfloor}\kappa\left(\frac{h}{b_n}\right)h+C_2\sum_{h=\lfloor n/2\rfloor+1}^{n-2}\kappa\left(\frac{h}{b_n}\right)
\end{aligned}
$$

The last inequality follows from Lemma 1, where $\widetilde{\rho}_S^X(h)$ is given by equation A10.2 and corresponds to a different estimator of the Spearman-autocorrelations. C_1 and C_2 are constant and independent of n and h. The last two terms of the above expression tend to 0 per our assumptions on the kernel, we therefore omit them from further algebraic transformations. It follows that

$$
\begin{aligned}
&\sum_{h=1}^{n-2}\kappa\left(\frac{h}{b_n}\right)\left(\mathbb{E}\left|\widetilde{\rho}_S^X(h)-\rho_S^X(h)\right|+\mathbb{E}\left|\widetilde{\rho}_S^Y(h)-\rho_S^Y(h)\right|\right) \\
&\leq \sum_{h=1}^{n-2}\kappa\left(\frac{h}{b_n}\right)\left(\left\|\widetilde{\rho}_S^X(h)-\rho_S^X(h)\right\|_2+\left\|\widetilde{\rho}_S^Y(h)-\rho_S^Y(h)\right\|_2\right) \\
&\leq \sum_{h=1}^{n-2}\kappa\left(\frac{h}{b_n}\right)\left(\left\|\frac{n-h-2}{n-h+1}U_n(h^{\rho_S^X(h)})-\rho_S^X(h)\right\|_2+\left\|\frac{n-h-2}{n-h+1}U_n(h^{\rho_S^Y(h)})-\rho_S^Y(h)\right\|_2+C_3\frac{1}{n-h}\right)
\end{aligned}
$$

Here $\|.\|_p$ denotes the usual L_p-norm $\|X\|_p=(\mathbb{E}\,|X|)^{1/p}$, and $U_n(h^{\rho_S^X(h)})$ denotes the U-statistic estimator of the Spearman-autocorrelations (equation A10.1), for which the algebraic equality A7 holds with $\widetilde{\rho}_S^X(h)$. Again C_3 ist independent of n and h and the term involving the constant C_3 tends to 0 per our assumptions on the kernel function. We now use the Hoeffding decomposition of the U-statistic estimator for the Spearman autocorrelations (see e.g. section 2.1 in Fischer et al., 2016a), which is given by

$$
\begin{aligned}
U_n(h^{\rho_S^X(h)}) = \rho_S^X(h) &+ \frac{3}{n-h}\sum_{i=1}^{n-h} g_1\left((X_i,X_{i+h})\right) \\
&+ \frac{3}{\binom{n-h}{2}}\sum_{1\leq i<j\leq n-h} g_2\left((X_i,X_{i+h}),(X_j,X_{j+h})\right) \\
&+ \frac{1}{\binom{n-h}{3}}\sum_{1\leq i<j<k\leq n-h} g_3\left((X_i,X_{i+h}),(X_j,X_{j+h}),(X_k,X_{k+h})\right)
\end{aligned}
$$

with

$$
g_1\left((x_1,x_{1+h})\right)=\mathbb{E}_{X_2,X_{2+h}}\mathbb{E}_{X_3,X_{3+h}}h^{\rho_S}\left((x_1,x_{1+h}),(X_2,X_{2+h}),(X_3,X_{3+h})\right)-\rho_S^X(h)
$$

$$g_2\left((x_1,x_{1+h}),(x_2,x_{2+h})\right) = \mathbb{E}_{X_3,X_{3+h}} h^{\rho_S}\left((x_1,x_{1+h}),(x_2,x_{2+h}),(X_3,X_{3+h})\right) - \rho_S^X(h) - g_1\left((x_i,x_{i+h})\right) - g_1\left((x_j,x_{j+h})\right)$$

$$\begin{aligned} g_3\left((x_1,x_{1+h}),(x_2,x_{2+h}),(x_3,x_{3+h})\right) = & h^{\rho_S}\left((x_1,x_{1+h}),(x_2,x_{2+h}),(x_3,x_{3+h})\right) - \rho_S^X(h) \\ & - g_1\left((x_1,x_{1+h})\right) - g_1\left((x_2,x_{2+h})\right) - g_1\left((x_3,x_{3+h})\right) \\ & - g_2\left((x_1,x_{1+h}),(x_2,x_{2+h})\right) - g_2\left((x_1,x_{1+h}),(x_3,x_{3+h})\right) \\ & - g_2\left((x_2,x_{2+h}),(x_3,x_{3+h})\right) \end{aligned}$$

where $h^{\rho_S}\left((x_1,x_{1+h}),(x_2,x_{2+h}),(x_3,x_{3+h})\right)$ is given by equation A3.1. This leads to

$$\begin{aligned} & \sum_{h=1}^{n-2} \kappa\left(\frac{h}{b_n}\right)\left(\left\|\frac{n-h-2}{n-h+1}U_n(h^{\rho_S^X(h)}) - \rho_S^X(h)\right\|_2 + \left\|\frac{n-h-2}{n-h+1}U_n(h^{\rho_S^Y(h)}) - \rho_S^Y(h)\right\|_2\right) \\ \leq & \sum_{h=1}^{n-2}\left[\kappa\left(\frac{h}{b_n}\right)\frac{n-h-2}{n-h+1}\left(\left\|\frac{3}{n-h}\sum_{i=1}^{n-h} g_1\left((X_i,X_{i+h})\right)\right\|_2\right.\right. \\ & + \left\|\frac{3}{\binom{n-h}{2}}\sum_{1\leq i<j\leq n-h} g_2\left((X_i,X_{i+h}),(X_j,X_{j+h})\right)\right\|_2 \\ & + \left\|\frac{1}{\binom{n-h}{3}}\sum_{1\leq i<j<k\leq n-h} g_3\left((X_i,X_{i+h}),(X_j,X_{j+h}),(X_k,X_{k+h})\right)\right\|_2 \\ & + \left\|\frac{3}{n-h}\sum_{i=1}^{n-h} g_1\left((Y_i,Y_{i+h})\right)\right\|_2 \\ & + \left\|\frac{3}{\binom{n-h}{2}}\sum_{1\leq i<j\leq n-h} g_2\left((Y_i,Y_{i+h}),(Y_j,Y_{j+h})\right)\right\|_2 \\ & \left. + \left\|\frac{1}{\binom{n-h}{3}}\sum_{1\leq i<j<k\leq n-h} g_3\left((Y_i,Y_{i+h}),(Y_j,Y_{j+h}),(Y_k,Y_{k+h})\right)\right\|_2\right) \\ & \left. + \kappa\left(\frac{h}{b_n}\right)\frac{3}{n-h-1}\left(\rho_S^X(h)+\rho_S^Y(h)\right)\right] \\ \leq & \sum_{h=1}^{\lfloor (n-3)/2\rfloor}\kappa\left(\frac{h}{b_n}\right)K\sqrt{\frac{h}{n-h}} + \sum_{h=\lfloor (n-3)/2\rfloor+1}^{n-2}\kappa\left(\frac{h}{b_n}\right)C + \sum_{i=1}^{n-2}\kappa\left(\frac{h}{b_n}\right)6\frac{h}{n}\left(\rho_S^X(h)+\rho_S^Y(h)\right) \\ \leq & \sum_{h=1}^{\lfloor (n-3)/2\rfloor}\kappa\left(\frac{h}{b_n}\right)K\sqrt{2\frac{h}{n}} + \sum_{h=\lfloor (n-3)/2\rfloor+1}^{n-2}\kappa\left(\frac{h}{b_n}\right)C + \sum_{i=1}^{n-2}\kappa\left(\frac{h}{b_n}\right)6\frac{h}{n}\left(\rho_S^X(h)+\rho_S^Y(h)\right) \end{aligned}$$

The penultimate inequality follows from Lemmas 2, 3 and 4. K and C are independent of h and n. This expression tends to 0 per our assumption on the kernel κ and the bandwidth b_n. Therefore, the estimator in equation 4.1 converges to the long-run variance in mean and hence

in probability (7.3.2 in Grimmett und Stirzaker, 2020).

□

Lemma 1. *For $h \leq \lfloor n/2 \rfloor$ it holds that*

$$|\widehat{\rho}_S(h) - \widetilde{\rho}_S(h)| \leq C\frac{h}{n}$$

where $\widehat{\rho}_S(h)$ is given by equation 4.2 and $\widetilde{\rho}_S(h)$ is given by equation A10.1.

Proof of Lemma 1. The algebraic relationship from eqation A7 holds for the following estimators of the Spearman-autocorrelation for lag h

$$U_n(h^{\rho_S^X(h)}) = \frac{1}{\binom{n-h}{3}} \sum_{1\leq i<j<k\leq n-h} h^{\rho_S}\left((X_i, X_{i+h}), (X_j, X_{j+h}), (X_k, X_{k+h})\right) \tag{A10.1}$$

$$\widetilde{\rho}_S^X(h) = \frac{\sum_{i=1}^{n-h}\left(R^X_{i|(1:n-h)} - \overline{R}^X_{(1:n-h)}\right)\left(R^X_{i+h|(1+h:n)} - \overline{R}^X_{(1+h:n)}\right)}{\sqrt{\sum_{i=1}^{n-h}\left(R^X_{i|(1:n-h)} - \overline{R}^X_{(1:n-h)}\right)^2 \sum_{i=h+1}^{n}\left(R^X_{i|(h+1:n)} - \overline{R}^X_{(h+1:n)}\right)^2}} \tag{A10.2}$$

Here $R_{j|(i:k)}$ is the rank of the variables X_j with respect to the set of random variables $\{X_i, \ldots, X_k\}$ with $i \leq j \leq k$. Algebraic transformations lead to

$$\widehat{\rho}_S(h) - \widetilde{\rho}_S(h) =$$

$$\underbrace{\frac{12}{n(n^2-1)}\sum_{i=1}^{n-h} R_i^X R_{i+h}^X - \frac{12}{(n-h)((n-h)^2-1)}\sum_{i=1}^{n-h} R^X_{i|(1:n-h)} R^X_{i+h|(1+h:n)}}_{I}$$

$$\underbrace{-\frac{3(n+h)(n+1)^2}{n(n^2-1)} + \frac{3(n-h)((n-h)+1)^2}{(n-h)((n-h)^2-1)}}_{II} + \underbrace{\frac{6(n+1)}{n(n^2-1)}\left(\sum_{i=1}^{h} R_i^X \sum_{i=n-h+1}^{n} R_i^X\right)}_{III}$$

Further algebraic transformations reveal

$$|I| \leq C_1\frac{h}{n}$$
$$|II| \leq C_2\frac{h}{n}$$
$$|III| \leq C_3\frac{h}{n}$$

where C_1, C_2 and C_3 are independent of h and n. These inequalities hold for $h = 1, \ldots, n-2$ for the first and second term and for $h \leq \lfloor n/2 \rfloor$ for the third term.

□

Lemma 2. *Let $(X_i)_{i\in\mathbb{Z}}$ be a strictly stationary, absolutely regular process with absolutely continuous marginal distribution and mixing coefficients β_k satisfying equation 3.A. Then*

$$\left\| \frac{3}{n-h} \sum_{i=1}^{n-h} g_1\left((X_i, X_{i+h})\right) \right\|_2 \leq K\sqrt{\frac{h}{n-h}} \qquad \text{for } h \leq \lfloor (n-1)/2 \rfloor$$

where K is independent of h and n.

Proof of Lemma 2. $g_1\left((X_i, X_{i+h})\right)_{i\in\mathbb{Z}}$ is a measurable function of $(X_i, X_{i+h})_{i\in\mathbb{Z}}$ and bounded. If $(X_i)_{i\in\mathbb{Z}}$ is absolutely reguar, the same holds for $(X_i, X_{i+h})_{i\in\mathbb{Z}}$ for any $h > 0$ and the same summation conditions apply to the mixing coefficients of $(X_i, X_{i+h})_{i\in\mathbb{Z}}$, such as equation 3.A (for $(\mathbf{Z}(h)_i)_{i\in\mathbb{Z}} = (X_i, X_{i+h})_{i\in\mathbb{Z}}$ it holds that $\beta_{k,\mathbf{Z}(h)} \leq \beta_{k-h}$ for $k \geq h+1$, where $\beta_{k,\mathbf{Z}(h)}$ refer to the β-mixing coefficients of $(\mathbf{Z}(h)_i)_{i\in\mathbb{Z}}$ and β_k refer to the mixing coefficients of $(X_i)_{i\in\mathbb{Z}}$). It follows from the inequality of Davydov (1970) for some $\delta > 0$

$$\left|\mathbb{E}g_1\left((X_i, X_{i+h})\right) g_1\left((X_{i+k}, X_{i+k+h})\right)\right| \leq c \cdot \beta_{k,\mathbf{Z}(h)}^{\frac{\delta}{2+\delta}}$$

Thus

$$\begin{aligned}
\left\| \frac{3}{n-h} \sum_{i=1}^{n-h} g_1\left((X_i, X_{i+h})\right) \right\|_2^2 =& \mathbb{E}\left(\frac{3}{n-h} \sum_{i=1}^{n-h} g_1\left((X_i, X_{i+h})\right) \right)^2 \\
\leq& \frac{9}{(n-h)^2} \sum_{i,j=1}^{n-h} \left|\mathbb{E}g_1\left((X_i, X_{i+h})\right) g_1\left((X_j, X_{j+h})\right)\right| \\
=& \frac{9}{(n-h)^2} \sum_{k=0}^{n-h-1} (n-h-k) \left|\mathbb{E}g_1\left((X_0, X_{0+h})\right) g_1\left((X_k, X_{k+h})\right)\right| \\
\leq& \frac{9}{n-h} \sum_{k=0}^{n-h-1} \left|\mathbb{E}g_1\left((X_0, X_{0+h})\right) g_1\left((X_k, X_{k+h})\right)\right| \\
\leq& \frac{9}{n-h} \sum_{k=0}^{n-h-1} c \cdot \beta_{k,\mathbf{Z}(h)}^{\frac{\delta}{2+\delta}}
\end{aligned}$$

Now we can use the trivial upper bound $\beta_{k,\mathbf{Z}(h)} \leq 1$ for $k \leq h$. And for $h \leq n-h-1$ respectively $h \leq \lfloor (n-1)/2 \rfloor$

$$\frac{9}{n-h} \sum_{k=0}^{n-h-1} c \cdot \beta_{k,\mathbf{Z}(h)}^{\frac{\delta}{2+\delta}} \leq \frac{9c}{n-h} \left(\sum_{k=0}^{h-1} 1 + \sum_{k=h}^{n-h-1} \beta_{k-h}^{\frac{\delta}{2+\delta}} \right) \leq \frac{9c}{n-h} \left(h + \sum_{k=0}^{\infty} \beta_k^{\frac{\delta}{2+\delta}} \right) \leq C \cdot \frac{h}{n-h}$$

For $h > \lfloor (n-1)/2 \rfloor$ we obtain a uniform bound

$$\frac{9}{n-h} \sum_{k=0}^{n-h-1} c \cdot \beta_{k,\mathbf{Z}(h)}^{\frac{\delta}{2+\delta}} \leq \frac{9c}{n-h}(n-h) = C$$

□

Lemma 3. *Let $(X_i)_{i\in\mathbb{Z}}$ be a strictly stationary, absolutely regular process with absolutely continuous marginal distribution and mixing coefficients β_k satisfying equation 3.A. It holds that*

$$\left\|\frac{3}{\binom{n-h}{2}}\sum_{1\le i<j\le n-h} g_2\left((X_i,X_{i+h}),(X_j,X_{j+h})\right)\right\|_2 \le K\frac{h}{n-h} \qquad \text{for } h\le\lfloor(n-2)/2\rfloor$$

where K is independent of h and n.

Proof of Lemma 3. g_2 is a measurable function and bounded. Let

$$J\left(X_{i_1},X_{i_2},X_{i_3},X_{i_4}\right)=g_2\left((X_{i_1},X_{i_1+h}),(X_{i_2},X_{i_2+h})\right)g_2\left((X_{i_3},X_{i_3+h}),(X_{i_4},X_{i_4+h})\right)$$

It follows from Lemma 1 of Yoshihara (1976) (see Lemma 2 in Dehling, 2006, for an alternative formulation of the lemma) that

$$\left|\mathbb{E}J\left(X_{i_1},X_{i_2},X_{i_3},X_{i_4}\right)\right|\le C\cdot\beta_{l,\mathbf{Z}(h)}^{\frac{\delta}{2+\delta}}$$

where $\beta_{k,\mathbf{Z}(h)}$ are the mixing coefficients of $(\mathbf{Z}(h)_i)_{i\in\mathbb{Z}}=(X_i,X_{i+h})_{i\in\mathbb{Z}}$, where we set $\beta_{k,\mathbf{Z}(h)}=1$ for $k\le h$, $l=\max\{i_{(2)}-i_{(1)},i_{(4)}-i_{(3)}\}$ with $i_{(j)}$ being the j-th smallest integer among $\{i_1,i_2,i_3,i_4\}$ $\left(i_{(1)}\le i_{(2)}\le i_{(3)}\le i_{(4)}\right)$, and C is independent of l and the indices. Now

$$\begin{aligned}
&\left(\left\|\frac{3}{\binom{n-h}{2}}\sum_{1\le i<j\le n-h} g_2\left((X_i,X_{i+h}),(X_j,X_{j+h})\right)\right\|_2\right)^2\\
&=\mathbb{E}\left(\frac{3}{\binom{n-h}{2}}\sum_{1\le i<j\le n-h} g_2\left((X_i,X_{i+h}),(X_j,X_{j+h})\right)\right)^2\\
&=\left(\frac{3}{\binom{n-h}{2}}\right)^2\sum_{1\le i_1<i_2\le n-h}\ \sum_{1\le i_3<i_4\le n-h}\mathbb{E}\left[g_2\left((X_{i_1},X_{i_1+h}),(X_{i_2},X_{i_2+h})\right)g_2\left((X_{i_3},X_{i_3+h}),(X_{i_4},X_{i_4+h})\right)\right]\\
&=\left(\frac{3}{\binom{n-h}{2}}\right)^2\sum_{1\le i_1<i_2\le n-h}\ \sum_{1\le i_3<i_4\le n-h}\mathbb{E}J\left(X_{i_1},X_{i_2},X_{i_3},X_{i_4}\right)\\
&=\left(\frac{3}{\binom{n-h}{2}}\right)^2\sum_{l=0}^{n-h-2}\ \sum_{\substack{1\le i_1<i_2\le n-h\\ 1\le i_3<i_4\le n-h\\ \max\{i_{(2)}-i_{(1)},i_{(4)}-i_{(3)}\}=l}}\mathbb{E}J\left(X_{i_1},X_{i_2},X_{i_3},X_{i_4}\right)
\end{aligned}$$

The number of terms in the inner sum can be obtained via combinatorial arguments. For a fixed l, there are two possibilities: Only the difference of the two largest or the two smallest indices equals l, or both differences equal l.

In the first case, assuming $1\le l\le n-h-2$, the difference between the two largest or the two smallest summation indices equals l, while the difference of the other pair is always smaller. We can split this up into two more cases: $l=i_{(2)}-i_{(1)}$ or $i_{(4)}-i_{(3)}$.

For $l=i_{(2)}-i_{(1)}$, there are $(n-h-l-1)$ possibilities of choosing the smallest index $i_{(1)}$, permit-

ting up to one equality among the remaining indices, and the position of $i_{(2)}$ is fixed. For $i_{(1)}$ and $i_{(2)}$ fixed, we can choose the remaining indices so that there is at most one or no equality among $i_{(2)}$, $i_{(3)}$ and $i_{(4)}$. The number of possibilities to choose $i_{(3)}$ depends on the number of permitted equalities. For $i_{(1)}$, $i_{(2)}$ and $i_{(3)}$ fixed, the number of possibilities for $i_{(4)}$ are determined by the constraints $i_{(4)} - i_{(3)} < l$, $i_{(4)} \leq n - h$ and $i_{(4)} - i_{(2)} > 0$. In the case of one pair of indices being equal to another, there are two ways to obtain this index set in the sum above. In the case of all indices being distinct, there are 6 ways to obtain an index set that fulfils the conditions above. Therefore, the number of terms is

$$
\begin{aligned}
&2 \sum_{i=1}^{n-h-l-1} \sum_{k=0}^{n-h-(i+l)-1} \min\{l-1, n-h-(i+l+k)\} \\
&+ 2 \sum_{i=1}^{n-h-l-1} \sum_{k=1}^{n-h-(i+l)} \min\{l, n-h-(i+l+k)+1\} \\
&+ 2 \sum_{i=1}^{n-h-l-2} \sum_{k=1}^{n-h-(i+l)-1} \min\{l-1, n-h-(i+l+k)\}
\end{aligned}
$$

The terms where the upper bound of the summation index of the first sum is smaller than 1 should be evaluated as 0. For the case of $l = i_{(4)} - i_{(3)}$ the number of terms is exactly the same which can be found via analogous arguments. Therefore, the number of terms for the asymmetrical case (the maximum is obtained for one pair of indices) is

$$
\begin{aligned}
4\Bigg(&\sum_{i=1}^{n-h-l-1} \sum_{k=0}^{n-h-(i+l)-1} \min\{l-1, n-h-(i+l+k)\} \\
+ &\sum_{i=1}^{n-h-l-1} \sum_{k=1}^{n-h-(i+l)} \min\{l, n-h-(i+l+k)+1\} \\
+ &\sum_{i=1}^{n-h-l-2} \sum_{k=1}^{n-h-(i+l)-1} \min\{l-1, n-h-(i+l+k)\}\Bigg) \leq 12(n-h-1)^2 l
\end{aligned}
$$

In the symmetrical case of $l = i_{(2)} - i_{(1)} = i_{(4)} - i_{(3)}$, there are $n - h - 2l$ ways to fix $i_{(1)}$ and $i_{(2)}$ is fixed implicitly. There are then $n - h - (i_{(1)} + 2l) + 1$ possibilities to choose $i_{(3)}$, $i_{(4)}$ is fixed implicitly. Again, in the case of three distinct integers there are 2 ways to obtain a set of indices fulfilling these conditions in the sum above, and in the case of four distinct integers there are six ways. The number of terms is given by

$$
\sum_{i=1}^{n-h-2l} [2(n-h-(i+2l)+1) + 4(n-h-(i+2l))] \leq 12(n-h-1)^2
$$

This term should be evaluated as 0 for $n - h - 2l \leq 0$. For the case of $l = 0$, the only relevant cases are $i_{(1)} = i_{(2)} < i_{(3)} = i_{(4)}$. There are $n - h - 1$ possibilities to fix $i_{(1)}$ and simultaneously $i_{(2)}$, and with that there are $(n - h - i_{(1)})$ possibilities to fix $i_{(3)}$ and hence $i_{(4)}$. Therefore, for $l = 0$, the number of terms is

$$\sum_{i=1}^{n-h-1} (n-h-i) \leq (n-h-1)^2$$

It follows

$$\left(\frac{3}{\binom{n-h}{2}}\right)^2 \sum_{l=0}^{n-h-2} \sum_{\substack{1\leq i_1<i_2\leq n-h \\ 1\leq i_3<i_4\leq n-h \\ \max\{i_{(2)}-i_{(1)}, i_{(4)}-i_{(3)}\}=l}} \mathbb{E}J\left(X_{i_1}, X_{i_2}, X_{i_3}, X_{i_4}\right)$$

$$\leq \left(\frac{3}{\binom{n-h}{2}}\right)^2 \left[C(n-h-1)^2 + C(n-h-1)^2 \sum_{l=1}^{n-h-2} (1+l)\beta_{l,\mathbf{Z}(h)}^{\frac{\delta}{2+\delta}}\right]$$

now for $h \leq \lfloor (n-2)/2 \rfloor$ we obtain

$$\left(\frac{3}{\binom{n-h}{2}}\right)^2 \left[C(n-h-1)^2 + C(n-h-1)^2 \sum_{l=1}^{n-h-2} (1+l)\beta_{l,\mathbf{Z}(h)}^{\frac{\delta}{2+\delta}}\right]$$

$$\leq \left(\frac{3}{\binom{n-h}{2}}\right)^2 \left[C(n-h-1)^2 + C(n-h-1)^2 \left(\sum_{l=1}^{h}(1+l) + \sum_{l=h+1}^{n-h-2}(1+l)\beta_{l-h}^{\frac{\delta}{2+\delta}}\right)\right] \leq c\frac{h^2}{(n-h)^2}$$

and so

$$\left\|\frac{3}{\binom{n-h}{2}} \sum_{1\leq i<j\leq n-h} g_2\left((X_i, X_{i+h}), (X_j, X_{j+h})\right)\right\|_2 \leq K\frac{h}{n-h}$$

For $h > \lfloor (n-2)/2 \rfloor$ we obtain a uniform bound

$$\left(\frac{3}{\binom{n-h}{2}}\right)^2 \left[C(n-h-1)^2 + C(n-h-1)^2 \sum_{l=1}^{n-h-2} (1+l)\beta_{l,\mathbf{Z}(h)}^{\frac{\delta}{2+\delta}}\right]$$

$$\leq \left(\frac{3}{\binom{n-h}{2}}\right)^2 \left[C(n-h-1)^2 + C(n-h-1)^2(n-h-2)\left(1+\frac{(n-h-1)}{2}\right)\right] \leq c$$

□

Lemma 4. *Let $(X_i)_{i\in\mathbb{Z}}$ be a strictly stationary, absolutely regular process with absolutely continuous marginal distribution and mixing coefficients β_k satisfying equation 3.A. It holds that*

$$\left\|\frac{1}{\binom{n-h}{3}}\sum_{1\leq i<j<k\leq n-h} g_3\left((X_i,X_{i+h}),(X_j,X_{j+h}),(X_k,X_{k+h})\right)\right\|_2 \leq K\frac{h}{n-h} \qquad \text{for } h\leq \lfloor (n-3)/2\rfloor$$

where K is independent of h and n.

Proof of Lemma 4. g_3 is measurable and bounded, as h^{ρ_S}, g_1 and g_2 are measurable and bounded. Let

$$\begin{aligned}&J\left(X_{i_1},X_{i_2},X_{i_3},X_{i_4},X_{i_5},X_{i_6}\right)\\ &= g_3\left((X_{i_1},X_{i_1+h}),(X_{i_2},X_{i_2+h}),(X_{i_3},X_{i_3+h})\right) g_3\left((X_{i_4},X_{i_4+h}),(X_{i_5},X_{i_5+h}),(X_{i_6},X_{i_6+h})\right)\end{aligned}$$

and let $\{i_{(1)},i_{(2)},i_{(3)},i_{(4)},i_{(5)},i_{(6)}\}=\{i_1,i_2,i_3,i_4,i_5,i_6\}$ with $i_{(1)}\leq i_{(2)}\leq i_{(3)}\leq i_{(4)}\leq i_{(5)}\leq i_{(6)}$. It follows from Lemma 1 of Yoshihara (1976) that

$$\left|\mathbb{E}J\left(X_{i_1},X_{i_2},X_{i_3},X_{i_4},X_{i_5},X_{i_6}\right)\right|\leq C\cdot\beta_{l,\mathbf{Z}(h)}^{\frac{\delta}{2+\delta}}$$

Here $\beta_{k,\mathbf{Z}(h)}$ refer to the mixing coefficients of $(\mathbf{Z}(h)_i)_{i\in\mathbb{Z}}=(X_i,X_{i+h})_{i\in\mathbb{Z}}$, which we set to 1 for $k\leq h$, and $l=\max\{i_{(2)}-i_{(1)},i_{(6)}-i_{(5)}\}$, where $i_{(j)}$ is the j-th largest integer among $\{i_1,i_2,i_3,i_4,i_5,i_6\}$. Now

$$\begin{aligned}&\left(\left\|\frac{1}{\binom{n-h}{3}}\sum_{1\leq i<j<k\leq n-h} g_3\left((X_i,X_{i+h}),(X_j,X_{j+h}),(X_k,X_{k+h})\right)\right\|_2\right)^2\\ &=\mathbb{E}\left(\frac{1}{\binom{n-h}{3}}\sum_{1\leq i<j<k\leq n-h} g_3\left((X_i,X_{i+h}),(X_j,X_{j+h}),(X_k,X_{k+h})\right)\right)^2\\ &=\left(\frac{1}{\binom{n-h}{3}}\right)^2\sum_{1\leq i_1<i_2<i_3\leq n-h}\ \sum_{1\leq i_4<i_5<i_6\leq n-h}\mathbb{E}J\left(X_{i_1},X_{i_2},X_{i_3},X_{i_4},X_{i_5},X_{i_6}\right)\\ &=\left(\frac{1}{\binom{n-h}{3}}\right)^2\sum_{l=0}^{n-h-3}\ \sum_{\substack{1\leq i_1<i_2<i_3\leq n-h\\ 1\leq i_4<i_5<i_6\leq n-h\\ \max\{i_{(2)}-i_{(1)},i_{(6)}-i_{(5)}\}=l}}\mathbb{E}J\left(X_{i_1},X_{i_2},X_{i_3},X_{i_4},X_{i_5},X_{i_6}\right)\end{aligned}$$

The number of terms in the inner sum for a fixed l can be obtained via combinatorial arguments. The maximum value for l is $n-h-3$, because of the conditions on the summation indices, and the minimum value for l is 0. For a fixed l, there are 2 possibilities: Only the difference of the two largest or the two smallest integers equals l, or both.
In the first case, assuming $1\leq l\leq n-h-3$, the difference of the two largest or the two smallest summation indices equals l, while the difference of the other is always smaller. We can split this up into two more cases: $l=i_{(2)}-i_{(1)}$ and $l=i_{(6)}-i_{(5)}$. For $l=i_{(2)}-i_{(1)}$: there are either $n-h-l-2$, $n-h-l-3$ or $n-h-l-4$ possibilities of choosing the smallest index

$i_{(1)}$, depending on how many equalities are allowed among the remaining indices. $i_{(2)}$ is fixed. For $i_{(3)}$, $i_{(4)}$ and $i_{(5)}$ the number of possible choices has to accommodate enough positions for the respective remaining indices. Finally, the choices for $i_{(6)}$ are determined by the constraints $i_{(6)} - i_{(5)} < l$, $i_{(6)} \leq n - h$ and $i_{(6)} - i_{(4)} > 0$. There are 20, 6 and 2 ways to obtain an index sextuple with no, one and, respectively, two equalities among them in the sum above. Therefore, the number of terms for this case is

$$2 \cdot (I + II + III) + 2 \cdot (IV + V + VI + VII) + 2 \cdot (V + VI) + 2 \cdot VIII$$

with

$$I: \sum_{i=1}^{n-h-l-2} \sum_{j=0}^{n-h-(i+l)-2} \sum_{k=1}^{n-h-(i+j+l)-1} \sum_{m=0}^{n-h-(i+j+k+l)-1} \min\{l-1, n-h-(i+l+j+k+m)\}$$

$$II: \sum_{i=1}^{n-h-l-2} \sum_{j=0}^{n-h-(i+l)-2} \sum_{k=1}^{n-h-(i+j+l)-1} \sum_{m=1}^{n-h-(i+j+k+l)} \min\{l, n-h-(i+l+j+k+m)+1\}$$

$$III: \sum_{i=1}^{n-h-l-2} \sum_{j=1}^{n-h-(i+l)-1} \sum_{k=0}^{n-h-(i+j+l)-1} \sum_{m=1}^{n-h-(i+j+k+l)} \min\{l, n-h-(i+l+j+k+m)+1\}$$

$$IV: \sum_{i=1}^{n-h-l-3} \sum_{j=0}^{n-h-(i+l)-3} \sum_{k=1}^{n-h-(i+j+l)-2} \sum_{m=1}^{n-h-(i+j+k+l)-1} \min\{l-1, n-h-(i+l+j+k+m)\}$$

$$V: \sum_{i=1}^{n-h-l-3} \sum_{j=1}^{n-h-(i+l)-2} \sum_{k=0}^{n-h-(i+j+l)-2} \sum_{m=1}^{n-h-(i+j+k+l)-1} \min\{l-1, n-h-(i+l+j+k+m)\}$$

$$VI: \sum_{i=1}^{n-h-l-3} \sum_{j=1}^{n-h-(i+l)-2} \sum_{k=1}^{n-h-(i+j+l)-1} \sum_{m=0}^{n-h-(i+j+k+l)-1} \min\{l-1, n-h-(i+l+j+k+m)\}$$

$$VII: \sum_{i=1}^{n-h-l-3} \sum_{j=1}^{n-h-(i+l)-2} \sum_{k=1}^{n-h-(i+j+l)-1} \sum_{m=1}^{n-h-(i+j+k+l)} \min\{l, n-h-(i+l+j+k+m)+1\}$$

$$VIII: \sum_{i=1}^{n-h-l-4} \sum_{j=1}^{n-h-(i+l)-3} \sum_{k=1}^{n-h-(i+j+l)-2} \sum_{m=1}^{n-h-(i+j+k+l)-1} \min\{l-1, n-h-(i+l+j+k+m)\}$$

If the upper bound of the first summation index of a sum in any of the terms above is smaller than 1 the expression should be evaluated as 0. By an argument of symmetry, the number of terms for $l = i_{(6)} - i_{(5)}$ is exactly the same, so the number of terms for the asymmetrical case (the maximum l is attained either between the two smallest or the two largest indices) is

$$\begin{aligned} &2 \cdot [2 \cdot (I + II + III) + 2 \cdot (IV + V + VI + VII) + 2 \cdot (V + VI) + 2 \cdot VIII] \\ &\leq 40 \cdot (n-h-1)^2 (n-h-2)^2 l \end{aligned}$$

In the symmetrical case of $l = i_{(6)} - i_{(5)} = i_{(2)} - i_{(1)}$, there are either $n - h - 2l - 1$, $n - h - 2l - 2$ or $n - h - 2l - 3$ ways to fix $i_{(1)}$, depending on how many equalities are permitted among the remaining indices. The possible cases include allowing for $i_{(2)} = i_{(3)}$ and $i_{(4)} = i_{(5)}$, or only

allowing for either one of those equalities, or allowing for $i_{(3)} = i_{(4)}$. As before, sextuples with no, one or two equalities can be obtained in 20, 6 or 2 ways, respectively. We obtain

$$IX: \sum_{i=1}^{n-h-2l-1} \sum_{j=0}^{n-h-l-(i+l)-1} \sum_{k=1}^{n-h-l-(i+j+l)} \sum_{m=0}^{n-h-l-(i+j+k+l)} 1$$

$$X: \sum_{i=1}^{n-h-2l-2} \sum_{j=0}^{n-h-l-(i+l)-2} \sum_{k=1}^{n-h-l-(i+j+l)-1} \sum_{m=1}^{n-h-l-(i+j+k+l)} 1$$

$$XI: \sum_{i=1}^{n-h-2l-2} \sum_{j=1}^{n-h-l-(i+l)-1} \sum_{k=0}^{n-h-l-(i+j+l)-1} \sum_{m=1}^{n-h-l-(i+j+k+l)} 1$$

$$XII: \sum_{i=1}^{n-h-2l-2} \sum_{j=1}^{n-h-l-(i+l)-1} \sum_{k=1}^{n-h-l-(i+j+l)} \sum_{m=0}^{n-h-l-(i+j+k+l)} 1$$

$$XIII: \sum_{i=1}^{n-h-2l-3} \sum_{j=1}^{n-h-l-(i+l)-2} \sum_{k=1}^{n-h-l-(i+j+l)-1} \sum_{m=1}^{n-h-l-(i+j+k+l)} 1$$

If the upper bound of the first summation index of a sum in any of the above expressions is smaller than 1 the expression should be evaluated as 0. The total number of terms for this case (the maximum l is attained for both the two smallest and the two largest indices) is

$$2 \cdot IX + 4 \cdot X + 6 \cdot XI + 4 \cdot XII + 4 \cdot XIII \leq 20 \cdot (n-h-1)^2(n-h-2)^2$$

All the considerations above are valid for $1 \leq l \leq n-h-3$. For the special case of $l = 0$, it holds that $i_{(1)} = i_{(2)}$ and $i_{(5)} = i_{(6)}$. The remaining indices $i_{(3)}$ and $i_{(4)}$ can be distinct or equal to one another. For the number of such terms we obtain

$$XIV: \sum_{i=1}^{n-h-2} \sum_{j=1}^{n-h-i-1} \sum_{k=0}^{n-h-(i+j)-1} \sum_{m=1}^{n-h-(i+j+k)} 1$$

$$XV: \sum_{i=1}^{n-h-3} \sum_{j=1}^{n-h-i-2} \sum_{k=1}^{n-h-(i+j)-1} \sum_{m=1}^{n-h-(i+j+k)} 1$$

If the upper bound on the first summation index of a sum in any of the above expressions is smaller than 1 the expression should be evaluated as 0. The total number of terms for $l = 0$ is

$$XIV + XV \leq 2 \cdot (n-h-1)^2(n-h-2)^2$$

So

$$\left(\frac{1}{\binom{n-h}{3}}\right)^2 \sum_{l=0}^{n-h-3} \sum_{\substack{1\le i_1<i_2<i_3\le n-h\\ 1\le i_4<i_5<i_6\le n-h\\ \max\{i_{(2)}-i_{(1)},i_{(6)}-i_{(5)}\}=l}} \mathbb{E}J\left(X_{i_1},X_{i_2},X_{i_3},X_{i_4},X_{i_5},X_{i_6}\right)$$

$$\le \left(\frac{1}{\binom{n-h}{3}}\right)^2 \left[C\cdot 2\cdot(n-h-1)^2(n-h-2)^2 + C\cdot 40\cdot(n-h-1)^2(n-h-2)^2 \sum_{i=1}^{n-h-3}(1+l)\beta_{l,\mathbf{Z}(h)}^{\frac{\delta}{2+\delta}}\right]$$

now for $h \le \lfloor (n-3)/2 \rfloor$ we obtain

$$\left(\frac{1}{\binom{n-h}{3}}\right)^2 \left[C\cdot 2\cdot(n-h-1)^2(n-h-2)^2 + C\cdot 40\cdot(n-h-1)^2(n-h-2)^2 \sum_{l=1}^{n-h-3}(1+l)\beta_{l,\mathbf{Z}(h)}^{\frac{\delta}{2+\delta}}\right]$$

$$\le \left(\frac{1}{\binom{n-h}{3}}\right)^2 \left[2C(n-h-1)^2(n-h-2)^2 + 40C(n-h-1)^2(n-h-2)^2 \left(\sum_{l=1}^{h}(1+l) + \sum_{l=h+1}^{n-h-3}(1+l)\beta_{l-h}^{\frac{\delta}{2+\delta}}\right)\right]$$

$$\le c\frac{h^2}{(n-h)^2}$$

and so

$$\left\|\frac{1}{\binom{n-h}{3}} \sum_{1\le i<j<k\le n-h} g_3\left((X_i,X_{i+h}),(X_j,X_{j+h}),(X_k,X_{k+h})\right)\right\|_2 \le K\frac{h}{n-h}$$

For $h > \lfloor (n-3)/2 \rfloor$ we obtain a uniform bound

$$\left(\frac{1}{\binom{n-h}{3}}\right)^2 \left[C\cdot 2\cdot(n-h-1)^2(n-h-2)^2 + C\cdot 40\cdot(n-h-1)^2(n-h-2)^2 \sum_{i=1}^{n-h-3}(1+l)\beta_{l,\mathbf{Z}(h)}^{\frac{\delta}{2+\delta}}\right]$$

$$\le \left(\frac{1}{\binom{n-h}{3}}\right)^2 \left[2C(n-h-1)^2(n-h-2)^2 + 40C(n-h-1)^2(n-h-2)^2(n-h-3)\left(1+\frac{n-h-2}{2}\right)\right]$$

$$\le c$$

□

Corollary 3. *Let $(X_i, Y_i)_{i\in\mathbb{Z}}$ be a bivariate, strictly stationary, absolutely regular process with absolutely continuous marginal distributions and β-mixing coefficients β_k satisfying equation 3.A. Let κ be a kernel function satisfying Assumption 1 (Assumption 1 in de Jong und Davidson (2000)) and b_n be a non-decreasing sequence with $b_n \to \infty$ and $b_n = o(n^{1/2})$. Let κ and b_n also satisfy equation 4.A. Under the assumption of pairwise dependence between $(X_i)_{i\in\mathbb{Z}}$ and $(Y_i)_{i\in\mathbb{Z}}$ with $\rho_S, \tau \neq 0$, the test based on the test statistics*

$$T_{\rho_S} = \frac{\sqrt{n}\widehat{\rho}_S}{\widehat{\sigma}^2} \tag{5.1}$$

$$T_{\tau} = \frac{\sqrt{n}\widehat{\tau}}{\frac{4}{9}\widehat{\sigma}^2} \tag{5.2}$$

with $\widehat{\sigma}^2$ from equation 4.1 is consistent.

Proof of Corollary 3. Under the alternative $\rho_S, \tau \neq 0$ we know that the respective test statistic is still asymptotically normally distributed from Theorem 2 in Dehling (2006), but the long-run variance differs from the case of pairwise independence. The long-run variance estimator $\widehat{\sigma}^2$ from equation 4.1 still converges to the long-run variance under the Null-hypothesis σ_0^2 (equation 3.1 and 3.2), no pairwise independence is needed for the consistency. We now look at the consistency of the test regarding Spearman's Rho, but the result for Kendall's Tau can be obtained completely analogously. We rewrite the test statistic in the following way

$$T_{\rho_S} = \frac{\sqrt{n}\widehat{\rho}_S}{\widehat{\sigma}^2} = \frac{\sqrt{n}(\widehat{\rho}_S - \rho_1)}{\widehat{\sigma}^2} + \frac{\sqrt{n}\rho_1}{\widehat{\sigma}^2} \tag{A11}$$

Where ρ_1 refers to the Spearman correlation of the pairs X_i and Y_i. From Slutsky's Lemma and Theorem 2 in Dehling (2006) it follows that the first term converges in distribution and is hence stochastically bounded

$$\frac{\sqrt{n}(\widehat{\rho}_S - \rho_1)}{\widehat{\sigma}^2} \xrightarrow{D} \mathcal{N}\left(0, \frac{\sigma_1^2}{\sigma_0^2}\right)$$

Here σ_0^2 refers to the long-run variance of the estimator under the Null hypothesis (A5.2) and σ_1^2 refers to the long-run variance of the estimator under the alternative, which is given by

$$\sigma_1^2 = \mathbb{E}\left(g_1(X_1, Y_1)\right)^2 + 2\sum_{j>0} \operatorname{Cov}\left(g_1(X_1, Y_1), g_1(X_j, Y_j)\right)$$

with

$$
\begin{aligned}
g_1\left((x_1,y_1)\right) = 2\Bigg[& 1 - F_X(x_1) - F_Y(y_1) + F_X(x_1)F_Y(y_1) \\
& +1 - F_X(x_1) - F_Y(y_1) + F_X(x_1)F_Y(y_1) \\
& + \int_{-\infty}^{\infty}\int_{-\infty}^{\infty} \mathbf{1}_{(0,\infty)}(x_1-u)[1-F_Y(v)]dF_{X,Y}(u,v) \\
& + \int_{-\infty}^{\infty}\int_{-\infty}^{\infty} \mathbf{1}_{(0,\infty)}(y_1-v)[1-F_X(u)]dF_{X,Y}(u,v) \\
& + \int_{-\infty}^{\infty} F_{X,Y}(x_1,v)dF_Y(v) \\
& + \int_{-\infty}^{\infty} F_{X,Y}(u,y_1)dF_X(u)\Bigg] - 3 - \rho_1
\end{aligned}
$$

For $\rho_1 \neq 0$ the second term in equation 14 diverges in probability, which implies the consistency of the test:

$$
\lim_{n\to\infty} \mathbb{P}\left(|T_{\rho_S}| \geq z_{1-\alpha/2} \middle| \mathcal{H}_1 : \rho_S = \rho_1\right) = \infty
$$

for any $\alpha > 0$. □

References

Davydov, Y. A. (1970). The Invariance Principle for Stationary Processes. *Theory of Probability & Its Applications*, *15*(3), 487–498. https://doi.org/10.1137/1115050

Dehling, H. (2006). Limit Theorems for Dependent U-statistics. *Dependence in Probability and Statistics* (S. 65–86). Springer, New York, NY. https://doi.org/10.1007/0-387-36062-X_3

de Jong, R. M. und Davidson, J. (2000). Consistency of Kernel Estimators of Heteroscedastic and Autocorrelated Covariance Matrices. *Econometrica*, *68*(2), 407–423.

Fischer, S., Fried, R. und Wendler, M. (2016a). Multivariate generalized linear-statistics of short range dependent data. *Electronic Journal of Statistics*, *10*(1), 646–682. https://doi.org/10.1214/16-EJS1124

Grimmett, G. und Stirzaker, D. (2020). *Probability and random processes.* Oxford University Press.

Nelsen, R. B. (1992). On measures of association as measures of positive dependence. *Statistics & Probability Letters*, *14*(4), 269–274. https://doi.org/10.1016/0167-7152(92)90056-B

van der Vaart, A. W. (1998). *Asymptotic Statistics.* Cambridge University Press. https://doi.org/10.1017/CBO9780511802256

Yoshihara, K. I. (1976). Limiting behavior of U-statistics for stationary, absolutely regular processes. *Zeitschrift für Wahrscheinlichkeitstheorie und Verwandte Gebiete*, *35*(3), 237–252. https://doi.org/10.1007/BF00532676
